# Association between receiving the *Aksi Bergizi* Social Behavioral Change Communication (SBCC) intervention and hygiene behaviors among secondary school students in Padang, Indonesia

**DOI:** 10.7717/peerj.19256

**Published:** 2025-04-07

**Authors:** Ricvan Dana Nindrea, Wit Wichaidit

**Affiliations:** 1 Department of Medicine, Faculty of Medicine, Universitas Negeri Padang, Bukittinggi, Indonesia; 2 Department of Epidemiology, Faculty of Medicine, Prince of Songkla University, Hat Yai, Thailand

**Keywords:** Adolescent, Hygiene behaviors, Health education

## Abstract

**Background:**

The Government of Indonesia and UNICEF introduced the *Aksi Bergizi* Social Behavioral Change Communication (SBCC) intervention to promote healthy eating and hygiene behaviors among adolescents. However, no systematic assessment of the program’s effect has been made. This study aims to assess the association between exposure to the *Aksi Bergizi* nutrition promotion program and hand, oral, and nail hygiene behaviors among secondary school students in Padang, Indonesia.

**Methods:**

We conducted a school-based cross-sectional study in Padang Municipality, Indonesia, collecting data from 253 students attending *Aksi Bergizi* target schools and 253 students from non-target schools using a self-administered questionnaire. We compared hygiene behaviors between students in the two groups using descriptive statistics and multivariable logistic regression with adjustment for demographic and socioeconomic characteristics.

**Results:**

All students reported brushing their teeth at least twice per day, so there was no observable difference regarding oral hygiene. However, we found that students in target schools were significantly more likely than those in non-target schools to always use soap when washing their hands both before eating (75% *vs.* 21%; Adjusted odds ratio (OR) = 6.03; 95% confidence interval (CI) [3.96–9.19]) and after using the toilet (74% *vs.* 21%; Adjusted OR = 5.74, 95% CI [3.78–8.72]). However, there was no statistically significant difference with regard to nail hygiene, *i.e*., cutting nails at least once per week.

**Conclusion:**

We found differences between target and non-target schools regarding self-reported handwashing but no differences in nail-clipping. The findings of this study have implications for stakeholders in infectious diseases and nutrition. Future studies should consider ways to reduce social desirability bias and increase the generalizability of the study findings.

## Introduction

Among adolescents, hygienic behaviours play a critical role in maintaining overall health and preventing the spread of infectious diseases (Jatrana et al., 2021; Singh et al., 2023). Hygiene refers to the practice of maintaining the cleanliness of body parts. The most common type of hygiene studied in public health is hand hygiene, which includes hand washing with soap and water as well as the use of hand rubs (*e.g.*, hand sanitizers) (UNICEF, 2022). Hand hygiene is universally recognized as a fundamental practice for preventing gastrointestinal and respiratory infections (Aiello et al., 2008). Oral hygiene refers to the practices that maintain the cleanliness and health of the mouth, teeth, and gums. This includes regular brushing and flossing of teeth, using mouthwash, and visiting the dentist for check-ups and cleanings (NIDCR, 2024). Poor oral hygiene after meals can lead to dental caries, gum diseases, and other health issues (Duangthip & Chu, 2020; Santhosh et al., 2023). Nail hygiene refers to the practices and behaviors that keep the fingernails and toenails clean, healthy, and free from harmful microorganisms (CDC, 2024). This includes regularly trimming nails, cleaning under them, and avoiding habits like biting nails or using unclean tools that can introduce germs or cause damage (CDC, 2024; Wu & Lipner, 2020). However, despite their importance, oral and nail hygiene practices are often overlooked as components of hygiene (Wu & Lipner, 2020), even among adolescents (Su et al., 2024). To promote hygiene and other health behaviors, various actors in health have applied Social Behavioral Change Communication (SBCC) tools. SBCC refers to the “systematic application of theory-based, research-driven communication strategies” (USAID, 2019) to influence social conditions and modify individual behaviors (McKee, Becker-Benton & Bockh, 2014).

Indonesia is a middle-income country in Southeast Asia where eating with hands is a common cultural practice, making hand hygiene promotion essential for reducing the risk of infectious diseases (Han et al., 2020). Health promotion programs in Indonesia that focus on adolescent nutrition may also include hygiene promotion. Despite this inclusion, hygiene behaviors are not as thoroughly described in the evaluation of these programs as dietary behaviors (Loveday et al., 2014). The Indonesian government, in collaboration with UNICEF, has implemented the *Aksi Bergizi* (“Nutrition Action”) program in 2022 in selected secondary schools nationwide. The program aimed to promote proper nutrition among adolescents based on SBCC principles (Ministry of Health of Indonesia, 2019b; Ministry of Health of Indonesia, 2019a; UNICEF, 2019), but also included hand, oral, and nail hygiene promotion activities. Information regarding hand, oral, and nail hygienes among students in schools that did and did not receive the *Aksi Bergizi* program can provide basic information and empirical evidence for stakeholders in adolescent health and infectious diseases. Therefore, the objective of this study is to assess the association between exposure to the *Aksi Bergizi* nutrition promotion program and hand, oral, nail hygiene behaviors among secondary school students in Padang, Indonesia.

## Materials & Methods

### Study design and setting

We conducted a school-based cross-sectional study in Padang Municipality, West Sumatra Province, Indonesia.

### Study participants and sample size calculation

The study participants were secondary school students aged 12-15 years in Padang Municipality, West Sumatra Province, Indonesia. Sample size calculation for the study was based on the main objective of comparing dietary habits between students of the target *vs.* non-target schools. We hypothesized that 59% of students in schools that received the *Aksi Bergizi* program had good knowledge of dietary habits (p1 = 0.59) compared to 42% of students in schools without the program (p2 = 0.42) (Davis et al., 2009; Ndagire, Muyonga & Nakimbugwe, 2019). With a 95% confidence level and 80% power, we calculated the sample size for comparing two proportions to be 253 students per group, resulting in a total of 506 students.

### Exposure: *Aksi Bergizi* program activities

The *Aksi Bergizi* nutrition promotion program included a series of activities based on SBCC for nutritional education and health promotion. The health promotion component included hand, oral, and nail hygiene promotion. The program mandated teachers from the school or healthcare staff from a local *puskesmas* (health centers) with demonstrating proper handwashing techniques and educating the students on the importance of regular brushing for oral health and the need for regular nail care to prevent infections. Teachers and healthcare staff also provided visual aids and distributed hygiene kits containing soap, toothpaste, toothbrushes, and nail clippers to encourage students to behavioral adoption and maintenance (Ministry of Health of Indonesia, 2019b; Ministry of Health of Indonesia, 2019a; UNICEF, 2019). The activities were held on the first week of every month. At the time of data collection, the Aksi Bergizi activities had been ongoing at target schools for approximately 18 months.

### Outcomes: self-reported hygiene behaviors

We developed the hygiene behavior questions based on the *Aksi Bergizi* modules. The questionnaire included six questions across three domains: hand hygiene, oral hygiene, and nail hygiene, focusing on activities within the past 30 days. The questionnaire included three questions on hand hygiene: “*How often did you wash your hands before eating?*”, “*How often did you wash your hands after using the toilet or latrine?*”, and “*How often did you use soap when washing your hands?*”. Answer choices ranged from “*Never*” to “*Always*”. The questionnaire also included two questions on oral hygiene: “*How many times per day did you usually clean or brush your teeth?*” and “*How often did you use toothpaste that contains fluoride when you cleaned or brushed your teeth?*”. Answer choices ranged from “*Never*” to “*Every day, more than twice per day*”. The questionnaire included one question on nail hygiene: “*How often did you clip your nails?*” and the answer choices ranged from “*Never*” to “*One time per week*”.

### Study instrument

Our study instrument was a self-administered paper and pen questionnaire. We designed the questionnaire in English and Bahasa Indonesia by adapting existing instruments. We conducted a pilo*t*-test of the study instrument in Solok Regency, which is one of the regencies/cities in West Sumatra Province. Further revision of the questionnaire was carried out through an iterative process. We also assessed the validity and reliability of the instruments during this period. The questions we used underwent validity and reliability testing, resulting in a Cronbach’s alpha value of >0.7. The final questionnaire contained 9 sections, including: (A) Demographic characteristics; (B) Exposure (Aksi Bergizi nutrition promotion program); (C) Behavioral drivers for dietary and health; (D) Dietary habits; (E) Personal Hygiene; (F) Perception of Non Communicable Diseases (NCDs); (G) Physical Activity; (H) Alcohol, Tobacco, and Substance Use, and; (I) Reproductive Health and Prevention of Sexually Transmitted Diseases (STDs). Each questionnaire took approximately 20 min to complete. The English translation of the instrument can be found in the Supplementary Information section.

### Sampling methodology

The secondary school database was obtained from the Padang Municipality Education Office, West Sumatra Province, Indonesia. The sampling technique used in this study was multistage stratified clustered sampling. In the first stage, the secondary school database was obtained from the Padang Municipality Education Office, West Sumatra Province, Indonesia. A stratum was created based on secondary schools being targeted and non-targeted for the *Aksi Bergizi* program. Two schools were then randomly selected from each group. There were four secondary schools (all public) that were targeted for the *Aksi Bergizi* program. Among the four schools, only two schools were in the junior high school level, and the investigators selected both schools for the study. There were 39 secondary schools that were not targeted for the program, and the investigators selected two junior high schools with travel time of at least 45 min from the target schools in order to avoid the spill-over effect. In each school, we performed a stratified random sampling of classrooms to select two classrooms per grade level using the list of classrooms provided by the school. To avoid any potential repercussions, we hereby refrain from disclosing the names of the schools.

### Data collection

We scheduled an appointment with the selected secondary schools to determine a feasible time and date for data collection in the selected classrooms. We then asked the principal or the teacher in charge to introduce the investigators to the students in the classrooms. The investigators informed the students about the study and requested their verbal informed consent. We requested and obtained a waiver of written informed consent and parental consent from our Institutional Review Boards (IRBs) in order to help us reassume the participants of their confidentiality. When we requested the waivers, we explained to the IRB that the study questionnaire contained sensitive issues, and that requesting parental permission might prevent participants with the outcomes of interest from participation and potentially introduce selection bias. The information and consent processes were conducted in a group setting within the selected classes, but we allowed for individual questions and answers during the recruitment process. After the students expressed verbal consent, we directly distributed the study questionnaires to the students and asked them to start filling out the questionnaires. We also ensured that no teacher was present in the classroom at the time. At the end of the data collection period, the students placed their questionnaire in an opaque envelope provided by the investigators, and placed the envelope in a locked box in front of the classroom or at a designated location. We collected data from 9 November 2023 to 20 December 2023.

### Data management

We opened the secured box in a private location and performed data entry on the paper questionnaires using the KoboToolbox platform, which uploaded the entered data to a password-protected server. For each questionnaire, two investigators performed data entry separately, and the principal investigator (RDN) checked for discrepancies between the two versions with regard to the unique identification number (ID) and other values, checked the original questionnaire, and made corrections accordingly.

### Data analysis

We used descriptive statistics to describe the characteristics of the study participants. For the association between receiving *Aksi Bergizi* intervention and hygiene behavior, we used bivariate descriptive analyses as well as bivariable and multivariable logistic regression analyses. In multivariable analyses, we adjusted for the student’s age and sex as well as the parents’ education as proxies for family socioeconomic status, based on the findings of previous studies (Jatrana et al., 2021; Wichaidit et al., 2019). We did not include missing values in our analyses. We conducted all analyses at 95% level of confidence.

### Ethical considerations

This study received ethical approval from the ethics committee of the Faculty of Medicine at Prince Songkla University (Approval No. REC.66-248-18-2).

## Results

A total of 253 students from the *Aksi Bergizi* target schools and 253 students from the *Aksi Bergizi* non-target schools participated in our study (response rate = 100%). There were significant socio-demographic differences between participants target and non-target schools regarding age distribution, the education level of the participants’ father, and household monthly income (Table 1). Students in target schools self-reported higher adherence to hand hygiene at key moments for hand hygiene (before eating, after using the toilet or latrine) than students at non-target schools (Table 2). Students from target schools were also significantly more likely to report adherences to oral hygiene and nail hygiene than students from non-target schools.

**Table 1:** Characteristics of the study participants in *Aksi Bergizi* target and non-target schools.

**Characteristic**	***Aksi Bergizi* nutrition** **promotion program**		***P*-value**
	**Target (*n* = 253)**	**Non target (*n* = 253)**	
**Sex**			
Male	106 (41.9%)	111 (43.9%)	0.719
Female	147 (58.1%)	142 (56.1%)	
**Age**			
12 years	12 (4.7%)	22 (8.7%)	0.036
13 years	72 (28.5%)	90 (35.6%)	
14 years	115 (45.5%)	88 (34.8%)	
15 years	54 (21.3%)	53 (20.9%)	
**Ethnicity**			
Minangnese	146 (57.7%)	167 (66.0%)	<0.001
Javanese	35 (13.8%)	57 (22.5%)	
Bataknese	8 (3.2%)	0 (0%)	
Sundanese	5 (2.0%)	0 (0%)	
Others	59 (23.3%)	29 (11.5%)	
**Father’s occupation**			
Civil servant/state enterprise	54 (21.3%)	74 (29.2%)	<0.001
Private sector employee	92 (36.4%)	79 (31.2%)	
Small-scale vendors/service providers	42 (16.6%)	30 (11.9%)	
Business owner/entrepreneur	26 (10.3%)	10 (4.0%)	
Laborer/manual workers	18 (7.1%)	60 (23.7%)	
Agriculture/ fishery	19 (7.5%)	0 (0%)	
Independent professions (*e.g*., lawyers, architects)	2 (0.8%)	0 (0%)	
**Father’s education**			
Junior high school	38 (15.0%)	65 (25.7%)	<0.001
Senior high school	86 (34.0%)	93 (36.8%)	
Vocational certificate	1 (0.4%)	0 (0%)	
Associate’s degree	70 (27.7%)	26 (10.3%)	
Bachelor’s degree	51 (20.2%)	69 (27.3%)	
Higher than bachelor’s degree	7 (2.8%)	0 (0%)	
**Mother’s occupation**			
Housewife	173 (68.4%)	199 (78.7%)	0.036
Civil servant/ state enterprise	15 (5.9%)	12 (4.7%)	
Private sector employee	22 (8.7%)	12 (4.7%)	
Small-scale vendors/ service providers	39 (15.4%)	30 (11.9%)	
Business owner/ entrepreneur	4 (1.6%)	0 (0%)	
**Mother’s education**			
Primary school	2 (0.8%)	0 (0%)	0.031
Junior high school	45 (17.8%)	63 (24.9%)	
Senior high school	101 (39.9%)	101 (39.9%)	
Vocational certificate	4 (1.6%)	10 (4.0%)	
Associate’s degree	80 (31.6%)	69 (27.3%)	
Bachelor’s degree	21 (8.3%)	10 (4.0%)	
**Household monthly income**			
<1,000,000 IDR	0 (0%)	0 (0%)	<0.001
1,000,000 to 2,000,000 IDR	31 (12.3%)	66 (26.1%)	
2,000,001 to 3,000,000 IDR	61 (24.1%)	77 (30.4%)	
3,000,001 to 4,000,000 IDR	95 (37.5%)	72 (28.5%)	
4,000,001 to 5,000,000 IDR	47 (18.6%)	33 (13.0%)	
5,000,001 to 6,000,000 IDR	19 (7.5%)	5 (2.0%)	
**Religion**			
Islam	240 (94.9%)	240 (94.9%)	0.999
Christianity	13 (5.1%)	13 (5.1%)	
**Body mass index (BMI)**			
Underweight (BMI < 18.5 kg/m^2^)	3 (1.2%)	2 (0.8%)	0.145
Normal (BMI 18.5–22.9 kg/m^2^)	245 (96.8%)	238 (94.1%)	
Overweight (23–24.9 kg/m^2^)	5 (2.0%)	13 (5.1%)	
Obesity (≥25 kg/m^2^)	0 (0%)	0 (0%)	
**Main sources of information about health behaviors**			
Television advertisements	63 (24.9%)	41 (16.2%)	0.003
Family and/or friends	131 (51.8%)	169 (66.8%)	
Social media platforms (*e.g.*, Facebook, X, Instagram, *etc*.)	59 (23.3%)	43 (17.0%)	

**Table 2:** Self-reported hygiene behaviors among students exposed to the *Aksi Bergizi* program and students not exposed to the program.

**Personal hygiene**	***Aksi Bergizi*** **nutrition** **promotion program**		***P*-value**
	**Target (*n* = 253)**	**Non target (*n* = 253)**	
**Hand hygiene**			
**During the past 30 days, how often did you wash your hands before eating?**			<0.001
Never	0 (0%)	0 (0%)	
Rarely	0 (0%)	0 (0%)	
Sometimes	0 (0%)	38 (15.0%)	
Most of the time	80 (31.6%)	161 (63.6%)	
Always	173 (68.4%)	54 (21.3%)	
**During the past 30 days, how often did you wash your hands after using the toilet or latrine?**			<0.001
Never	0 (0%)	0 (0%)	
Rarely	0 (0%)	0 (0%)	
Sometimes	1 (0.4%)	5 (2.0%)	
Most of the time	84 (33.3%)	194 (76.7%)	
Always	167 (66.3%)	54 (21.3%)	
**During the past 30 days, how often did you use soap when washing your hands?**			<0.001
Never	0 (0%)	0 (0%)	
Rarely	0 (0%)	30 (11.9%)	
Sometimes	14 (5.5%)	71 (28.2%)	
Most of the time	80 (31.6%)	97 (38.5%)	
Always	159 (62.8%)	54 (21.4%)	
**Oral hygiene**			
**During the past 30 days, how many times per day did you usually clean or brush your teeth?**			<0.001
Never	0 (0%)	0 (0%)	
Not every day	0 (0%)	0 (0%)	
Every day, once per day	0 (0%)	0 (0%)	
Every day, twice per day	201 (79.4%)	237 (93.7%)	
Every day, more than twice per day	52 (20.6%)	16 (6.3%)	
**During the past 30 days, how often did you use a toothpaste that contains fluoride when you cleaned or brushed your teeth?**			<0.001
Never	0 (0%)	0 (0%)	
Not every day	0 (0%)	0 (0%)	
Every day, once per day	0 (0%)	0 (0%)	
Every day, twice per day	198 (78.3%)	237 (93.7%)	
Every day, more than twice per day	55 (21.7%)	16 (6.3%)	
**Nail hygiene**			
**During the past 30 days, how often did you clip your nails?**			0.001
Never	0 (0%)	0 (0%)	
One time per week	207 (81.8%)	217 (85.8%)	
One time per two weeks	41 (16.2%)	20 (7.9%)	
One time per three weeks	5 (2.0%)	16 (6.3%)	
One time per four weeks or did not clip	0 (0%)	0 (0%)	

Students at *Aksi Bergizi* target schools were significantly more likely than students at the non-target schools to self-reported handwashing with soap at all times *vs.* the otherwise before eating (75% *vs.* 21%; Adjusted OR = 6.03; 95% CI [3.96–9.19]) (Table 3) and after using the toilet (74% *vs.* 21%; Adjusted OR = 5.74, 95% CI [3.78–8.72]). However, there was no statistically significant association between school group and clipping nails.

**Table 3:** Association between attending *Aksi Bergizi* target *vs.* non-target schools and self-reported hygiene behaviors.

**Behavior**	**No outcome**	**Outcome**	**Unadjusted OR (95% CI)**	Adjusted OR (95% CI)^*^
**Handwashing before eating**	**Does not always wash hands or does not always use soap during handwashing**	**Always wash hands and always uses soap**		
*Aksi Bergizi* non-target schools	199 (78.7%)	54 (21.3%)	*Reference*	*Reference*
*Aksi Bergizi* target schools	94 (32.1%)	159 (74.6%)	**6.23 (4.20, 9.25)**	**6.03 (3.96, 9.19)**
**Handwashing after toilet use**	**Does not always wash hands or does not always use soap during handwashing**	**Always wash hands and always uses soap**		
*Aksi Bergizi* non-target schools	199 (78.7%)	54 (21.3%)	*Reference*	*Reference*
*Aksi Bergizi* target schools	96 (32.5%)	156 (74.3%)	**5.99 (4.04, 8.88)**	**5.74 (3.78, 8.72)**
**Nail hygiene**	**Cut nails less than once per week**	**Cut nails once per week**		
*Aksi Bergizi* non-target schools	36 (14.2%)	217 (85.8%)	*Reference*	*Reference*
*Aksi Bergizi* target schools	46 (18.2%)	207 (81.8%)	0.75 (0.46, 1.20)	0.64 (0.38, 1.08)

**Notes.**

* Adjusted for age and sex of the child, father’s education, and mother’s education

Bold numbers denote statistical significance at 95% level of confidence.

## Discussion

In this school-based cross-sectional study, we compared self-reported hygiene behaviors among students from schools that received the *Aksi Bergizi* nutrition promotion program with hygiene-related activities *vs.* students from schools that did not receive the program. We found universal compliance to oral hygiene. We also found significant differences regarding the probability of handwashing with soap before eating and after toilet use, but no statistically significant difference regarding nail hygiene behaviour.

The findings of our study regarding hand hygiene align with these previous studies (Jatrana et al., 2021; Rahman Zuthi et al., 2022). The additional contribution of our study findings may be the data pertaining to oral hygiene and nail hygiene behaviors, which were less commonly studied. In that regard, all of the students in our study, regardless of school type, reported brushing teeth at least twice per day. Although the participants self-administered the questionnaire, they might have feared being overseen by their peers and thus were less likely to self-report socially undesirable behaviors, and social desirability is common in self-reporting oral hygiene practices (AlGhamdi et al., 2020; Sanzone et al., 2013). Future studies should consider alternative ways to measure oral hygiene that may be less prone to social desirability, such as asking participants to click on the link for the questionnaire when they are home and safe from being overseen by peers.

While the study highlighted significant disparities in hygiene behaviors, several important aspects remained unknown or understudied. Firstly, previous studies have found gaps btween self-reported *vs.* observed hygiene behaviors (Hagiya et al., 2022; Seyed Nematian et al., 2017). Future studies should consider including a structured observation of hand hygiene behavior, similar to that in a previous study (Wichaidit et al., 2019). Furthermore, hygiene is influenced by disgust and social norms (Nizame et al., 2013; Wichaidit et al., 2019). We did not include measurement questions for these behavioral drivers in our study. Future studies should consider developing a theory of behavior change for the intervention, and collect data on potential drivers of hygiene behaviors and make pathway analyses according to the developed theory to achieve a more complete understanding of the observed differences.

The strength of our study was the high level of voluntary participation, which reduced selection bias from non-response. However, a number of limitations should be considered in the interpretation of the study findings. Firstly, the intervention was not randomized, and the cross-sectional design of the study precludes the ability to make inferences regarding program effectiveness. Secondly, hygiene behavior is deemed in many regions, including the study area, to be socially desirable. Thus, the influence of social desirability may be non-negligible in our study findings. Lastly, we conducted our study only among schools in Padang, West Sumatera. The findings of the study may not be generalizable to students and schools in other regions.

## Conclusions

We found that students in schools that received the *Aksi Bergizi* nutritional promotion program were more likely to self-report handwashing with soap before eating and after toilet use, but there was no significant difference regarding frequency of nail-clipping. Considering the role of hygiene in improving nutrition, the findings of this study may be relevant to stakeholders in both adolescent infectious diseases and adolescent nutrition. However, limitations regarding the cross-sectional design, social desirability, and limited generalizability should be considered in the interpretation of the study findings.

## Supplemental Information

10.7717/peerj.19256/supp-1 Supplemental Information 1 STROBE Checklist

10.7717/peerj.19256/supp-2 Supplemental Information 2 Dataset The raw data does not contain any personally identifiable information.

10.7717/peerj.19256/supp-3 Supplemental Information 3 Data Dictionary in English

10.7717/peerj.19256/supp-4 Supplemental Information 4 Questionnaire (CRF), English

10.7717/peerj.19256/supp-5 Supplemental Information 5 Questionnaire (CRF), Bahasa Indonesia

## Additional Information and Declarations

### Data Availability

The following information was supplied regarding data availability: The raw data are available in the Supplementary File.
