# Supplementary material for: Association between receiving the Aksi Bergizi Social Behavioral Change Communication (SBCC) intervention and hygiene behaviors among secondary school students in Padang, Indonesia
Source: PeerJ. 2025 Apr 7;13:e19256. doi: 10.7717/peerj.19256 (PMC11984468; doi:10.7717/peerj.19256)
Supplement: Supplemental Information 4 [file peerj-13-19256-s004.pdf]

QUESTIONNAIRE

Self-Reported Health Behaviors and Behavioral Drivers among Secondary School Students Attending Target and Non-Target Schools for the *Aksi Bergizi* Nutrition Promotion Program

Date: dd/mm/yyyy

Section A. Demographic characteristics

No	Questions	Answers (Please tick)
A1	Unique ID	[ ] [ ] [ ] [ ]
A2	Sex	[ ] 1. Male<br>[ ] 2. Female<br>[ ] 9. Refuse to answer
A3	Date of Birth	Day: [ ] [ ]<br>Month:[ ] [ ]<br>Year:[ ] [ ] [ ] [ ]
A4	Ethnicity	[ ] 1. Minangnese<br>[ ] 2. Javanese<br>[ ] 3. Bataknese<br>[ ] 4. Sundanese<br>[ ] 5. Others<br>[ ] 88. Don't know<br>[ ] 99. Refuse to answer
A5	Fathers' occupation	[ ] 1. Civil servant / state enterprise<br>[ ] 2. Private sector employee<br>[ ] 3. Small-scale vendors / service providers<br>[ ] 4. Business owner / entrepreneur<br>[ ] 5. Laborer / manual workers<br>[ ] 6. Retired / homemaker<br>[ ] 7. Agriculture / Fishery<br>[ ] 8. Independent professions (e.g., lawyers, architects)<br>[ ] 9. Unemployed<br>[ ] 99. Refuse to answer<br>[ ] 0. Deceased (***Skip to Question A7***)

A6	Fathers' education	[ ] 1. Never went to school<br>[ ] 2. Primary school<br>[ ] 3. Junior high school<br>[ ] 4. High school<br>[ ] 5. Vocational certificate<br>[ ] 6. Associate's degree<br>[ ] 7. Vocational diploma<br>[ ] 8. Bachelor's degree<br>[ ] 9. Higher than bachelor's degree<br>[ ] 88. Not sure<br>[ ] 99. Refuse to answer
A7	Mothers' occupation	[ ] 1. Housewife/ retired/ homemaker<br>[ ] 2. Civil servant / state enterprise<br>[ ] 3. Private sector employee<br>[ ] 4. Small-scale vendors / service providers<br>[ ] 5. Business owner / entrepreneur<br>[ ] 6. Laborer / manual workers<br>[ ] 7. Agriculture / Fishery<br>[ ] 8. Independent professions (e.g., lawyers, architects)<br>[ ] 9. Unemployed<br>[ ] 99. Refuse to answer<br>[ ] 0. Deceased (***Skip to Question A9***)
A8	Mothers' education	[ ] 1. Never went to school<br>[ ] 2. Primary school<br>[ ] 3. Junior high school<br>[ ] 4. High school<br>[ ] 5. Vocational certificate<br>[ ] 6. Associate's degree<br>[ ] 7. Vocational diploma<br>[ ] 8. Bachelor's degree<br>[ ] 9. Higher than bachelor's degree<br>[ ] 88. Not sure<br>[ ] 99. Refuse to answer
A9	Household Monthly Income	[ ] 1. No more than 1,000,000 IDR<br>[ ] 2. 1,000,000 to 2,000,000 IDR<br>[ ] 3. 2,000,001 to 3,000,000 IDR<br>[ ] 4. 3,000,001 to 4,000,000 IDR<br>[ ] 5. 4,000,001 to 5,000,000 IDR<br>[ ] 6. 5,000,001 to 6,000,000 IDR<br>[ ] 7. More than 6,000,000 IDR<br>[ ] 8. Not sure / Uncertain / Don't know<br>[ ] 9. Refuse to answer

A10	Religion	[ ] 1. Islam<br>[ ] 2. Buddhism<br>[ ] 3. Christianity<br>[ ] 4. Others<br>[ ] 9. Refuse to answer
A11	What is your approximate weight (in kg.)? (if don't know, write 888; if refused to answer, write 999)	[ ][ ][ ]
A12	What is your approximate height (in cm.)? (If don't know, write 888; if refused to answer, write 999)	[ ][ ][ ]
A13	What are the main media sources that you often encounter for obtaining information about health behaviors?	[ ] 1. Television advertisements<br>[ ] 2. Community events<br>[ ] 3. Informational brochures<br>[ ] 4. Family and/or friends<br>[ ] 5. Social media platforms (e.g., Facebook, Twitter, Instagram, etc)
A14	When getting and searching for health issues, how accurate do you think the information is?	[ ] 1. Very accurate<br>[ ] 2. Somewhat accurate<br>[ ] 3. Very inaccurate
A15	Social media provides helpful resources for health information	[ ] 1. Strongly agrre<br>[ ] 2. Agree<br>[ ] 3. Disagree<br>[ ] 4. Strongly disagree

Section B. Exposures: *Aksi Bergizi* nutrition promotion program

No	Questions	Answer
B1	Did you participate in the activity of *Aksi Bergizi* session on visual replicas food shown and matched to nutrional needs?	[ ] 0. No (skip to B3)<br>[ ] 1. Yes
B2	If yes, did you attend the following activities?	[ ] 1. Briefing<br>[ ] 2. Activity<br>[ ] 3. Discussion
B3	Did you participate in the activity of *Aksi Bergizi* session on personal hygiene and some examples of action?	[ ] 0. No (skip to B5)<br>[ ] 1. Yes
B4	If yes, did you attend the following activities?	[ ] 1. Briefing<br>[ ] 2. Activity<br>[ ] 3. Discussion
B5	Did you participate in the activity of *Aksi Bergizi* session on myths and facts about HIV and became the subject of discussion on HIV?	[ ] 0. No (skip to B7)<br>[ ] 1. Yes
B6	If yes, did you attend the following activities?	[ ] 1. Briefing<br>[ ] 2. Activity<br>[ ] 3. Discussion
B7	Did you participate in the activity of *Aksi Bergizi* session on the addictive substance poster functions as a trigger for discussion by presenting a third person as a form of self-projection regarding adolescents who are vulnerable to becoming drug users?	[ ] 0. No (skip to B9)<br>[ ] 1. Yes
B8	If yes, did you attend the following activities?	[ ] 1. Briefing<br>[ ] 2. Activity<br>[ ] 3. Discussion
B9	Did you participate in the activity of *Aksi Bergizi* session on NCDs illustrations, and the participants can get to know NCDs more scientifically?	[ ] 0. No (skip to B11)<br>[ ] 1. Yes

B10	If yes, did you attend the following activities?	[ ] 1. Briefing<br>[ ] 2. Activity<br>[ ] 3. Discussion
B11	Did you participate in the activity of *Aksi Bergizi* session on various examples of physical activity, and calculate various physical activities that are effective and that participants can easily do on a daily basis?	[ ] 0. No (skip to B13)<br>[ ] 1. Yes
B12	If yes, did you attend the following activities?	[ ] 1. Briefing<br>[ ] 2. Activity<br>[ ] 3. Discussion
B13	Which of the following would describe that you participated in the activities?	[ ] 1. Full (paying close attention, looking, and listening everything)<br>[ ] 2. Relatively high (talked to friends occasionally, but looking, and listening everything)

Section C. Behavioral drivers for dietary and health

No	Questions	Answer
**Cognitive biases**		
C1	I am easily influenced by the first piece of nutritional information I come across when making food choice	[ ] 1. Strongly disagree<br>[ ] 2. Disagree<br>[ ] 3. Agree<br>[ ] 4. Strongly agree
C2	To what extent are you influenced by health-related advertisements or endorsements, leading you to make choices that may not align with your best interests?	[ ] 1. Strongly disagree<br>[ ] 2. Disagree<br>[ ] 3. Agree<br>[ ] 4. Strongly agree
C3	How often do you seek out information or arguments that confirm your belief that smoking is harmful to your health?	[ ] 1. Never<br>[ ] 2. Rarely<br>[ ] 3. Sometimes<br>[ ] 4. Most of the time<br>[ ] 5. Always
C4	How likely are you to underestimate the health risks of alcohol consumption based on the fact that you know people who drink regularly without apparent negative consequences?	[ ] 1. Very unlikely<br>[ ] 2. Unlikely<br>[ ] 3. Likely<br>[ ] 4. Very likely
C5	How often do you let social or peer influences sway your decision not to	[ ] 1. Never

	consume substances, such as feeling pressured to use substances in certain social settings?	[ ] 2. Rarely<br>[ ] 3. Sometimes<br>[ ] 4. Most of the time<br>[ ] 5. Always
C6	To what extent do you prioritize immediate comfort or convenience over the potential long-term health benefits associated with physical activity?	[ ] 1. Not at all<br>[ ] 2. Slightly<br>[ ] 3. Moderate<br>[ ] 4. Completely
**Interest**		
C7	How interested are you in learning about the impact of dietary choices on your overall health and well-being?	[ ] 1. Not at all interested<br>[ ] 2. Not very interested<br>[ ] 3. Somewhat interested<br>[ ] 4. Very interested
C8	How excited are you about the potential health benefits and improved quality of life that come with not smoking?	[ ] 1. Not at all excited<br>[ ] 2. Not very excited<br>[ ] 3. Somewhat excited<br>[ ] 4. Very excited
C9	How motivated are you to reduce or eliminate your alcohol consumption for the sake of your health and well-being?	[ ] 1. Not at all motivated<br>[ ] 2. Not very motivated<br>[ ] 3. Somewhat motivated<br>[ ] 4. Very motivated
C10	How willing are you to seek support and resources to help you resist the temptation to use drugs?	[ ] 1. Not willing at all<br>[ ] 2. Not very willing<br>[ ] 3. Somewhat willing<br>[ ] 4. Very willing
C11	How curious are you about the benefits and improvements you could experience in your life by being physically active?	[ ] 1. Not curious at all<br>[ ] 2. Not very curious<br>[ ] 3. Somewhat curious<br>[ ] 4. Very curious
C12	How interested are you in learning about methods and practices to protect your reproductive health?	[ ] 1. Not at all interested<br>[ ] 2. Not very interested<br>[ ] 3. Somewhat interested<br>[ ] 4. Very interested
**Attitude**		
C13	How likely are you to believe that a healthy diet can contribute to overall well-being and prevent diseases?	[ ] 1. Very unlikely<br>[ ] 2. Unlikely<br>[ ] 3. Likely<br>[ ] 4. Very likely
C14	To what extent do you believe that not smoking is important for your health?	[ ] 1. Not important<br>[ ] 2. Slightly important<br>[ ] 3. Quite important

		[ ] 4. Very important
C15	How confident are you in your ability to resist the temptation to consume alcohol?	[ ] 1. Not committed<br>[ ] 2. Slightly committed<br>[ ] 3. Quite committed<br>[ ] 4. Very committed
C16	How enjoyable do you find a drug-free lifestyle compared to consuming drugs?	[ ] 1. Not enjoyable<br>[ ] 2. Slightly enjoyable<br>[ ] 3. Quite enjoyable<br>[ ] 4. Very enjoyable
C17	How important is it for you to incorporate regular physical activity into your daily routine?	[ ] 1. Not important<br>[ ] 2. Slightly important<br>[ ] 3. Quite important<br>[ ] 4. Very important
C18	How likely are you to seek regular check-ups and screenings to ensure your reproductive health is in good condition?	[ ] 1. Very unlikely<br>[ ] 2. Unlikely<br>[ ] 3. Likely<br>[ ] 4. Very likely
**Self-efficacy**		
C19	I am confident that I can maintain a healthy and balanced diet that includes fruits, and vegetables	[ ] 1. Strongly disagree<br>[ ] 2. Disagree<br>[ ] 3. Agree<br>[ ] 4. Strongly agree
C20	I am confident that I can limit my consumption of sugary drinks (e.g., soda, energy drinks) to one per week or less	[ ] 1. Strongly disagree<br>[ ] 2. Disagree<br>[ ] 3. Agree<br>[ ] 4. Strongly agree
C21	I am confident that I can resist the temptation to engage in unhealthy behaviors (e.g., excessive snacking, skipping exercise)	[ ] 1. Strongly disagree<br>[ ] 2. Disagree<br>[ ] 3. Agree<br>[ ] 4. Strongly agree
C22	I am confident that I can do jogging three days or more per week instead of bicycling at regular pace	[ ] 1. Strongly disagree<br>[ ] 2. Disagree<br>[ ] 3. Agree<br>[ ] 4. Strongly agree
C23	I am confident that I can do walking five days or more per week instead of sitting or laying down to watch television	[ ] 1. Strongly disagree<br>[ ] 2. Disagree<br>[ ] 3. Agree<br>[ ] 4. Strongly agree
C24	I am confident that I can quit smoking (if applicable) and remain smoke-free	[ ] 1. Strongly disagree<br>[ ] 2. Disagree<br>[ ] 3. Agree<br>[ ] 4. Strongly agree

C25	I am confident that I can manage my time effectively to prioritize and engage in healthy behaviors regularly	[ ] 1. Strongly disagree<br>[ ] 2. Disagree<br>[ ] 3. Agree<br>[ ] 4. Strongly agree
C26	I am confident that I can seek social support and engage in healthy activities with friends or family members	[ ] 1. Strongly disagree<br>[ ] 2. Disagree<br>[ ] 3. Agree<br>[ ] 4. Strongly agree
C27	I am confident that I can cope with stress or negative emotions without relying on alcohol and substance use as a coping mechanism	[ ] 1. Strongly disagree<br>[ ] 2. Disagree<br>[ ] 3. Agree<br>[ ] 4. Strongly agree
**Intent**		
C28	How determined are you to make changes in your dietary and health behaviors?	[ ] 1.Not determined at all<br>[ ] 2.Slightly determined<br>[ ] 3.Moderately determined<br>[ ] 4.Very determined
C29	How likely are you to actively pursue and incorporate healthy dietary and health behaviors into your daily life	[ ] 1. Very unlikely<br>[ ] 2. Unlikely<br>[ ] 3. Likely<br>[ ] 4. Very likely
C30	How committed are you to making long-term changes in your dietary and health behaviors?	[ ] 1. Not committed<br>[ ] 2. Slightly committed<br>[ ] 3. Quite committed<br>[ ] 4. Very committed
**Limited rationality**		
C31	To what extent do you rely on shortcuts or simplified thinking when making decisions about your dietary and health behaviors?	[ ] 1. Never<br>[ ] 2. Rarely<br>[ ] 3. Sometimes<br>[ ] 4. Most of the time<br>[ ] 5. Always
C32	How much do you tend to make decisions about your dietary and health behaviors based on limited information or incomplete understanding?	[ ] 1. Not at all<br>[ ] 2. Very little<br>[ ] 3. Moderately<br>[ ] 4. Quit a bit<br>[ ] 5. A lot
C32	Do you find it challenging to consider all available options and make fully informed choices regarding your dietary and health behaviors?	[ ] 1. Not at all<br>[ ] 2. Very little<br>[ ] 3. Moderately<br>[ ] 4. Quit a bit<br>[ ] 5. A lot

Section D. Dietary habits

No	Questions	Answers (Please tick)
note_ ffq	**Food Frequency Questionnaire**<br><br>How often do you eat the following food?	
D1	Rice (white rice)	[ ] 1. Never or less than once per month<br>[ ] 2. 1-3 times per month<br>[ ] 3. Once a week<br>[ ] 4. 2-4 times per week<br>[ ] 5. 5-6 times per week<br>[ ] 6. Once a day<br>[ ] 7. More than once a day<br>[ ] 99. Refuse to answer
D2	Refined wheat products (white bread, noodles)	[ ] 1. Never or less than once per month<br>[ ] 2. 1-3 times per month<br>[ ] 3. Once a week<br>[ ] 4. 2-4 times per week<br>[ ] 5. 5-6 times per week<br>[ ] 6. Once a day<br>[ ] 7. More than once a day<br>[ ] 99. Refuse to answer
D3	Coarse grain (brown rice)	[ ] 1. Never or less than once per month<br>[ ] 2. 1-3 times per month<br>[ ] 3. Once a week<br>[ ] 4. 2-4 times per week<br>[ ] 5. 5-6 times per week<br>[ ] 6. Once a day<br>[ ] 7. More than once a day<br>[ ] 99. Refuse to answer
D4	Whole grain wheat products (e.g., brown bread, whole wheat noodles)	[ ] 1. Never or less than once per month<br>[ ] 2. 1-3 times per month<br>[ ] 3. Once a week<br>[ ] 4. 2-4 times per week<br>[ ] 5. 5-6 times per week<br>[ ] 6. Once a day<br>[ ] 7. More than once a day

No	Questions	Answers (Please tick)
		[ ] 99. Refuse to answer
D5	Tubers (cassava, taro, white yams, white potato)	[ ] 1. Never or less than once per month<br>[ ] 2. 1-3 times per month<br>[ ] 3. Once a week<br>[ ] 4. 2-4 times per week<br>[ ] 5. 5-6 times per week<br>[ ] 6. Once a day<br>[ ] 7. More than once a day<br>[ ] 99. Refuse to answer
D6	Meat (beef, mutton)	[ ] 1. Never or less than once per month<br>[ ] 2. 1-3 times per month<br>[ ] 3. Once a week<br>[ ] 4. 2-4 times per week<br>[ ] 5. 5-6 times per week<br>[ ] 6. Once a day<br>[ ] 7. More than once a day<br>[ ] 99. Refuse to answer
D7	Poultry (duck, chicken)	[ ] 1. Never or less than once per month<br>[ ] 2. 1-3 times per month<br>[ ] 3. Once a week<br>[ ] 4. 2-4 times per week<br>[ ] 5. 5-6 times per week<br>[ ] 6. Once a day<br>[ ] 7. More than once a day<br>[ ] 99. Refuse to answer
D8a	Fish (raw, grilled, soup, not deep-fried)	[ ] 1. Never or less than once per month<br>[ ] 2. 1-3 times per month<br>[ ] 3. Once a week<br>[ ] 4. 2-4 times per week<br>[ ] 5. 5-6 times per week<br>[ ] 6. Once a day<br>[ ] 7. More than once a day<br>[ ] 99. Refuse to answer
D8b	Fresh seafood (e.g., clams, prawns, crabs, octopus)	[ ] 1. Never or less than once per month<br>[ ] 2. 1-3 times per month<br>[ ] 3. Once a week<br>[ ] 4. 2-4 times per week<br>[ ] 5. 5-6 times per week<br>[ ] 6. Once a day<br>[ ] 7. More than once a day

No	Questions	Answers (Please tick)
		[ ] 99. Refuse to answer
D9	Eggs	[ ] 1. Never or less than once per month<br>[ ] 2. 1-3 times per month<br>[ ] 3. Once a week<br>[ ] 4. 2-4 times per week<br>[ ] 5. 5-6 times per week<br>[ ] 6. Once a day<br>[ ] 7. More than once a day<br>[ ] 99. Refuse to answer
D10	Leafy green vegetables (e.g., Chinese cabbage, long bean, kale, spinach, yu choy, cucumber)	[ ] 1. Never or less than once per month<br>[ ] 2. 1-3 times per month<br>[ ] 3. Once a week<br>[ ] 4. 2-4 times per week<br>[ ] 5. 5-6 times per week<br>[ ] 6. Once a day<br>[ ] 7. More than once a day<br>[ ] 99. Refuse to answer
D11	Yellow or orange vegetables (e.g., pumpkin, sweet potatoes, carrots, ripened papaya)	[ ] 1. Never or less than once per month<br>[ ] 2. 1-3 times per month<br>[ ] 3. Once a week<br>[ ] 4. 2-4 times per week<br>[ ] 5. 5-6 times per week<br>[ ] 6. Once a day<br>[ ] 7. More than once a day<br>[ ] 9. Refuse to answer
D12	Soybean products (e.g., tofu)	[ ] 1. Never or less than once per month<br>[ ] 2. 1-3 times per month<br>[ ] 3. Once a week<br>[ ] 4. 2-4 times per week<br>[ ] 5. 5-6 times per week<br>[ ] 6. Once a day<br>[ ] 7. More than once a day<br>[ ] 99. Refuse to answer
D13	Preserved vegetables (e.g., canned pickled vegetables)	[ ] 1. Never or less than once per month<br>[ ] 2. 1-3 times per month<br>[ ] 3. Once a week<br>[ ] 4. 2-4 times per week<br>[ ] 5. 5-6 times per week

No	Questions	Answers (Please tick)
		[ ] 6. Once a day<br>[ ] 7. More than once a day<br>[ ] 99. Refuse to answer
D14	Fresh fruits	[ ] 1. Never or less than once per month<br>[ ] 2. 1-3 times per month<br>[ ] 3. Once a week<br>[ ] 4. 2-4 times per week<br>[ ] 5. 5-6 times per week<br>[ ] 6. Once a day<br>[ ] 7. More than once a day<br>[ ] 99. Refuse to answer
D15	Dairy products (fresh milk, powdered milk, boxed milk)	[ ] 1. Never or less than once per month<br>[ ] 2. 1-3 times per month<br>[ ] 3. Once a week<br>[ ] 4. 2-4 times per week<br>[ ] 5. 5-6 times per week<br>[ ] 6. Once a day<br>[ ] 7. More than once a day<br>[ ] 99. Refuse to answer
D16	Packaged snacks (e.g., corn puffs, Lay potato chips)	[ ] 1. Never or less than once per month<br>[ ] 2. 1-3 times per month<br>[ ] 3. Once a week<br>[ ] 4. 2-4 times per week<br>[ ] 5. 5-6 times per week<br>[ ] 6. Once a day<br>[ ] 7. More than once a day<br>[ ] 99. Refuse to answer
D17	Unpackaged snacks (donuts, french fries, roti, etc.)	[ ] 1. Never or less than once per month<br>[ ] 2. 1-3 times per month<br>[ ] 3. Once a week<br>[ ] 4. 2-4 times per week<br>[ ] 5. 5-6 times per week<br>[ ] 6. Once a day<br>[ ] 7. More than once a day<br>[ ] 99. Refuse to answer
D18	Sweetened drinks or condiments (soy milk, soft drinks, coke, coffee or tea with sugar, sweetened condensed milk)	[ ] 1. Never or less than once per month<br>[ ] 2. 1-3 times per month<br>[ ] 3. Once a week<br>[ ] 4. 2-4 times per week<br>[ ] 5. 5-6 times per week

No	Questions	Answers (Please tick)
		[ ] 6. Once a day<br>[ ] 7. More than once a day<br>[ ] 99. Refuse to answer
D19	Processed or ultra-processed foods (canned fish, sausages, canned vegetables, frozen food, etc.)	[ ] 1. Never or less than once per month<br>[ ] 2. 1-3 times per month<br>[ ] 3. Once a week<br>[ ] 4. 2-4 times per week<br>[ ] 5. 5-6 times per week<br>[ ] 6. Once a day<br>[ ] 7. More than once a day<br>[ ] 99. Refuse to answer
D20	Dessert (e.g., ice cream, cake, candy, cookies, sticky rice with mango, other sweets)	[ ] 1. Never or less than once per month<br>[ ] 2. 1-3 times per month<br>[ ] 3. Once a week<br>[ ] 4. 2-4 times per week<br>[ ] 5. 5-6 times per week<br>[ ] 6. Once a day<br>[ ] 7. More than once a day<br>[ ] 99. Refuse to answer
D21	Deep-fried meat or fish (fried, fish, fried chicken, fried beef)	[ ] 1. Never or less than once per month<br>[ ] 2. 1-3 times per month<br>[ ] 3. Once a week<br>[ ] 4. 2-4 times per week<br>[ ] 5. 5-6 times per week<br>[ ] 6. Once a day<br>[ ] 7. More than once a day<br>[ ] 99. Refuse to answer

Section E. Personal Hygiene

Please answer to what extent do you practice with the following statements?

No	Questions	Answer
E1	During the past 30 days, how often did you wash your hands before eating?	[ ] 1. Never<br>[ ] 2. Rarely<br>[ ] 3. Sometimes<br>[ ] 4. Most of the time<br>[ ] 5. Always
E2	During the past 30 days, how often did you wash your hands after using the toilet or latrine?	[ ] 1. Never<br>[ ] 2. Rarely<br>[ ] 3. Sometimes

		[ ] 4. Most of the time<br>[ ] 5. Always
E3	During the past 30 days, how often did you use soap when washing your hands?	[ ] 1. Never<br>[ ] 2. Rarely<br>[ ] 3. Sometimes<br>[ ] 4. Most of the time<br>[ ] 5. Always
E4	During the past 30 days, how many times per day did you usually clean or brush your teeth?	[ ] 1. Never<br>[ ] 2. Not every day<br>[ ] 3. Every day, once per day<br>[ ] 4. Every day, twice per day<br>[ ] 5. Every day, more than twice per day
E5	During the past 30 days, how often did you u use a toothpaste that contains fluoride when you cleaned or brushed your teeth?	[ ] 1. Never<br>[ ] 2. Not every day<br>[ ] 3. Every day, once per day<br>[ ] 4. Every day, twice per day<br>[ ] 5. Every day, more than twice per day
E6	During the past 30 days, how often did you clip your nails?	[ ] 1. Never<br>[ ] 2. One time per week<br>[ ] 3. One time per two weeks<br>[ ] 4. One time per three weeks<br>[ ] 5. One time per four weeks or did not clip

Section F. Perception of Non Communicable Diseases (NCDs)

Please indicate how much do you agree with the following statements:

No	Questions	Strongly agree	Agree	Disagree	Strongly Disagree
F1	I feel that I can effectively tell parents to do not smoke				
F2	I feel that I can remind my family member to maintain suitable bodyweight				
F3	I feel that I can remind my family member to do physical activities actively				

F4	I feel that I can effectively remind my family member to eat a healthy balanced diet (or) maintain an eating healthy balanced diet to prevent NCDs				
F5	I feel that I can effectively remind my family member to take prescribed drugs regularly, if they have either hypertension or hyperlipidemia or diabetes				

F6. In your opinion, how can young people help Indonesia fight NCDs?

[..]

Section G. Physical Activity

Think about all the **vigorous** activities that you did in the **last 7 days**. Vigorous physical activities refer to activities that take hard physical effort and make you breathe much harder than normal. Think only about those physical activities that you did for at least 10 minutes at a time?

G1. During the last 7 days, on how many days did you do vigorous physical activities like heavy lifting, digging, aerobics, or fast bicycling?

_____ days per week (range 1-7)

[ ] No vigorous physical activities → **Skip to question G3**

G2. How much time did you usually spend doing vigorous physical activities on one of those days?

_____ hours per day

_____ minutes per day

[ ] Don't know/ not sure

Think about all the **moderate** activities that you did in the **last 7 days**. **Moderate** activities refer to activities that take moderate physical effort and make you breathe somewhat harder than normal. Think only about those physical activities that you did for at least 10 minutes at a time.

G3. During the last 7 days, on how many days did you do moderate physical activities like carrying light loads, bicycling at a regular pace, or doubles tennis? Do not include walking.

_____ days per week (range 1-7)

[ ] No moderate physical activities → **Skip to question G5**

G4. How much time did you usually spend doing moderate physical activities on one of those days?

_____ hours per day

_____ minutes per day

[ ] Don't know/ not sure

Think about the time you spent **walking** in the **last 7 days**. This includes at work and at home, walking to travel from place to place, and any other walking that you have done solely for recreation, sport, exercise, or leisure.

G5. During the last 7 days, on how many days did you walk for at least 10 minutes at a time?

_____ days per week (range 1-7)

[ ] No walking → **Skip to question G7**

G6. How much time did you usually spend walking on one of those days?

_____ hours per day

_____ minutes per day

[ ] Don't know/ not sure

The last question is about the time you spent **sitting** on weekdays during the **last 7 days**. Include time spent at work, at home, while doing course work and during leisure time. This may include time spent sitting at a desk, visiting friends, reading, or sitting or lying down to watch television.

G7. During the last 7 days, how much time did you spend sitting on a week day?

_____ hours per day

_____ minutes per day

[ ] Don't know/ not sure

Section H. Alcohol, Tobacco, and Substance Use

H1. In your opinion, why do young people use addictive substances? Why do some of those who use addictive substances do so excessively, to the point where there is a substance use disorder?

[...]

H2. In your opinion, how can the young generation avoid using addictive substances?

[...]

Instructions Please mark ✓ **in the blank space that corresponds to your answer**

Alcohol Use	
H3. In your lifetime, have you ever drank alcohol (not including sipping or tasting in very small amount)?	[ ] 0) Never drank ***Skip to Question H8)***<br>[ ] 1) Yes<br>[ ] 88) Not sure ***(Skip to Question H8)***<br>[ ] 99) Refuse to answer ***(Skip to Question H8)***
H4. When did you **start** drinking alcohol for the first time?	Started drinking at age...............years<br>[ ] 88) Not sure<br>[ ] 99) Refuse to answer
H5. When was the most recent occasion that you drank (not including sipping or tasting in very small amount)?)	[ ] 1) More than 12 months ago<br>[ ] 2) More than 30 days ago but within past 12 months<br>[ ] 3) More than 1 week ago but within past 30 days<br>[ ] 4) Within the past 1 week
H6. If you wanted to drink alcohol, how long would it take you to buy/find a drink?	It would take...................... minutes<br>[ ] 77) More than 1 day<br>[ ] 88) Not sure<br>[ ] 99) Refuse to answer
H7. **In the past 30 days,** how many times did you drink?	[ ] 0) None<br>[ ] 1) 1-2 times<br>[ ] 2) 3-5 times<br>[ ] 3) 6-9 times

	[ ] 4) 10-19 times<br>[ ] 5) 20 times or more<br>[ ] 88) Not sure<br>[ ] 99) Refuse to answer
**Tobacco use**	
H8. In your lifetime, have you smoked more than 5 packs or 100 sticks of cigarettes?	[ ] 0) Never smoked ***(skip to Question H16)***<br>[ ] 1) Yes, but not more than 5 packs or 100 sticks<br>[ ] 2) Yes<br>[ ] 88) Not sure ***(skip to Question H16)***<br>[ ] 99) Refuse to answer ***(skip to Question H16)***
H9. How old were you when you ***started*** smoking entire cigarettes or other tobacco products	Started smoking at..............years of age<br>[ ] 88) Not sure<br>[ ] 99) Refuse to answer
H10. When was the most recent occasion when you smoked cigarette?	[ ] 1) More than 12 months ago ***(skip to Question H16)***<br>[ ] 2) More than 30 days ago but within past 12 months<br>[ ] 3) More than 1 week ago but within past 30 days<br>[ ] 4) Within the past 1 week<br>[ ] 88) Not sure<br>[ ] 99) Refuse to answer
H11. If you wanted to smoke a cigarette, how long would it take you to buy/find one?	It would take...................... minutes<br>[ ] More than 1 day<br>[ ] 88) Not sure<br>[ ] 99) Refuse to answer
H12. In the past 12 months, have you ever tried to quit smoking cigarettes?	[ ] 0) No<br>[ ] 1) Yes<br>[ ] 99) Refuse to answer
H13. In the past 12 months, have you ever stopped smoking cigarettes?	[ ] 0) No<br>[ ] 1) Yes, for ………….. days<br>[ ] 99) Refuse to answer
H14. In the past 30 days, how many days did you smoke?	[ ] 0) None ***(Skip to Question H16)***<br>[ ] 1) 1-2 days<br>[ ] 2) 3-5 days<br>[ ] 3) 6-9 days<br>[ ] 4) 10-19 days<br>[ ] 5) 20 days or more<br>[ ] 88) Not sure<br>[ ] 99) Refuse to answer

H15. <u>In the past 30 days</u>, on <u>the days that you smoked</u>, how many cigarettes did you use?	[ ] 1) Less than 1 per day<br>[ ] 2) 1 per day<br>[ ] 3) 2-5 per day<br>[ ] 4) 6-10 per day<br>[ ] 5) 11-20 per day<br>[ ] 6) More than 20 per day<br>[ ] 88) Not sure<br>[ ] 99) Refuse to answer
H16. In your lifetime, have you ever used an <u>electronic cigarette</u>?	[ ] 0) Never smoked <u>***(Skip to Question H22)***</u><br>[ ] 1) Yes<br>[ ] 99) Refuse to answer <u>***(Skip to Question H22)***</u>
H16a. If yes, how old were you when you first used an <u>electronic cigarette</u>?	a) I started when I was …………… years old<br>[ ] 88) Not sure<br>[ ] 99) Refuse to answer
H17. When was the most recent occasion when you used an <u>electronic cigarette</u>?	[ ] 1) More than 12 months ago <u>***(Skip to Question H22)***</u><br>[ ] 2) More than 30 days ago but within past 12 months<br>[ ] 3) More than 1 week ago but within past 30 days<br>[ ] 4) Within the past 1 week<br>[ ] 88) Not sure<br>[ ] 99) Refuse to answer
H18. <u>In the past 12 months</u>, have you ever tried to quit using electronic cigarette?	[ ] 0) No<br>[ ] 1) Yes<br>[ ] 99) Refuse to answer
H19. <u>In the past 12 months</u>, have you ever stopped using electronic cigarette?	[ ] 0) No<br>[ ] 1) Yes, for ………….. days<br>[ ] 99) Refuse to answer
H20. <u>In the past 30 days,</u> how many days did you use <u>electronic cigarette</u>?	[ ] 0) None <u>***(Skip to Question H22)***</u><br>[ ] 1) 1-2 days<br>[ ] 2) 3-5 days<br>[ ] 3) 6-9 days<br>[ ] 4) 10-19 days<br>[ ] 5) 20 days or more<br>[ ] 88) Not sure<br>[ ] 99) Refuse to answer
H21. <u>In the past 30 days</u>, on <u>the days that you used electronic cigarette</u>, how many times per day did you use <u>electronic</u>	[ ] 1) Less than 1 per day<br>[ ] 2) 1 per day<br>[ ] 3) 2-5 per day<br>[ ] 4) 6-10 per day

cigarette? cigarettes did you use? (one "TIME" consists of around 15 puffs)	[ ] 5) 11-20 per day<br>[ ] 6) More than 20 per day<br>[ ] 88) Not sure<br>[ ] 99) Refuse to answer

**Substance Use**						
**H22. Have you ever used the following drug or substance?**	**1) In your lifetime (if "No", go to the next row) or (if "Yes", go to the column to the right)**		**2) In the past 12 months (if "No", go to the next row) or (if "Yes", go to the column to the right)**		**3) In the past 30 days**	
	**No**	**Yes**	**No**	**Yes**	**No**	**Yes**
a. Analgesic (not as medication) (*parasetamol*)						
b. Antihistamine (not as medication) (*cetirizine*)						
c. Cough syrup (not as medication) (*obat batuk*)						
d. Anxiolytics (*obat anti depresi*)						
e. Sedatives (*obat penenang*)						
f. Energy drinks (*extra joss, M-150, kratingdaeng, hemaviton, lainnya*)						
g. *PRO* [procodyl, promethazine] (*obat anti mabok/ antimo*)						
h. *LEAN* [purple drank] (*racikan dari obat demam dengan dosis tinggi yang dicampur dengan minuman soda, es, dan juga permen sebagai penambah rasa*)						
i. Poppers (*produk kimia yang mengandung zat Amyl nitrite yang digunakan dengan cara dihirup*)						
j. Kratom leaf (*daun kratom, ketum, purik, sepat, kedamba, ithang, kakuan, thom, atau biak)*						
k. Kratom leaf tea mixed with other substance (*daun kratom, ketum, purik, sepat, kedamba, ithang, kakuan, thom, atau*						

biak yang dicampur dengan zat lain)						
l. Cannabis (*Ganja*)						
m. Opium (*Candu*)						
n. Ecstasy / Love Drug (*Inex*)						
o. Ketamine (*K khusus*)						
p. Heroin (*Putau*)						
q. Inhalants (paint thinner, glue, benzene) (*menghirup cairan tiner, lem, dan lainnya*)						
r. Methamphetamine (*Sabu*)						
s. Crystal methamphetamine (*Narkoba Kristal*)						

Section I. Reproductive Health and Prevention of Sexually Transmitted Diseases (STDs)

Please indicate how much do you agree with the following statements:

No	Questions	Strongly agree	Agree	Disagree	Strongly Disagree
I1	I do not want to give salaam when greeting someone who is HIV/AIDS positive				
I2	Adolescents who are HIV-positive should be banned from attending schools with HIV-negative students				
I3	Adolescents who are HIV-positive should not swim in a pool with HIV-negative students				
I4	Adolescents who are HIV-positive should not take part in sports competitions with HIV-negative students				

I5. If you have a friend who is HIV positive, you went swimming with them and someone said something against your friend using the pool. What would you do?

[……………………………………………………………………………]

<u>Instructions</u> Please mark ✓ **in the blank space that corresponds to your answer**

I6a. Have you ever had a romantic partner?	[ ] 0) No<br>[ ] 1) Yes<br>[ ] 9) Refuse to answer
I6b. To which gender are you attracted? (multiple answers allowed)	[ ] 1) Male (cisgender)<br>[ ] 2) Female (cisgender)<br>[ ] 3) Transgender female (*Bencong)*<br>[ ] 4) Transgender male (Tomboy*)*<br>[ ] 5) Neither male nor female<br>[ ] 6) I'm not sure to whom I am attracted<br>[ ] 7) I'm not attracted to any gender<br>[ ] 9) Refuse to answer
I7. What method of safe sex or prevention from STDs did you know? (More than 1 answer allowed)	[ ] 0) None<br>[ ] 1) Absitinence<br>[ ] 2) Avoiding activities high risk with mucosal tear<br>[ ] 3) Wore condom<br>[ ] 4) Suppository<br>[ ] 5) Taking Pre-exposure prophylaxis (PreP)<br>[ ] 6) Taking Post-exposure prophylaxis (PEP)<br>[ ] 7) Other methods, specify.......................<br>[ ] 88) Don't know / Not sure<br>[ ] 99) Refuse to answer
I8. Have you ever been taught about AIDS or HIV at school?	[ ] 0) Never<br>[ ] 1) Yes<br>[ ] 8) Don't know / Not sure<br>[ ] 9) Refuse to answer
I9. Have you ever been taught about birth control at school?	[ ] 0) Never<br>[ ] 1) Yes<br>[ ] 8) Don't know / Not sure<br>[ ] 9) Refuse to answer
