# Supplementary material for: Association between receiving the Aksi Bergizi Social Behavioral Change Communication (SBCC) intervention and hygiene behaviors among secondary school students in Padang, Indonesia
Source: PeerJ. 2025 Apr 7;13:e19256. doi: 10.7717/peerj.19256 (PMC11984468; doi:10.7717/peerj.19256)
Supplement: Supplemental Information 5 [file peerj-13-19256-s005.pdf]

**KUESIONER PENELITIAN**

**Perilaku Kesehatan yang Dilaporkan Secara Mandiri dan Pendorong Perilaku pada Siswa Sekolah Menengah yang Mengikuti Program Promosi *Aksi Bergizi* di Sekolah Target dan Non-Target**

Tanggal: Tanggal/Bulan/Tahun

## Bagian A. Karakteristik demografi

| No | Pertanyaan | Jawaban (Silahkan dicentang) |
|---|---|---|
| A1 | No ID | [ ] [ ] [ ] [ ] |
| A2 | Jenis kelamin | [ ] 1. Laki-laki<br>[ ] 2. Perempuan<br>[ ] 9. Menolak untuk menjawab |
| A3 | Tanggal lahir | Hari: [ ] [ ]<br>Bulan:[ ] [ ]<br>Tahun:[ ] [ ] [ ] [ ] |
| A4 | Etnis | [ ] 1. Minang<br>[ ] 2. Jawa<br>[ ] 3. Batak<br>[ ] 4. Sunda<br>[ ] 5. Lainnya<br>[ ] 88. Tidak tahu<br>[ ] 99. Menolak untuk menjawab |
| A5 | Pekerjaan ayah | [ ] 1. PNS<br>[ ] 2. Pegawai swasta<br>[ ] 3. Pedagang kecil<br>[ ] 4. Pemilik bisnis / pengusaha<br>[ ] 5. Buruh<br>[ ] 6. Pensiunan<br>[ ] 7. Bertani/ nelayan<br>[ ] 8. Profesional mandiri (pengacara, arsitek, lainnya)<br>[ ] 9. Tidak bekerja<br>[ ] 99. Menolak untuk menjawab<br>[ ] 0. Meninggal (***Lanjut ke pertanyaan A7***) |
| A6 | Fathers' education | [ ] 1. Tidak pernah sekolah<br>[ ] 2. Tamat SD<br>[ ] 3. Tamat SMP<br>[ ] 4. Tamat SMA<br>[ ] 5. Tamat kejuruan |

| | | |
|---|---|---|
| | | [ ] 6. Tamat D3<br>[ ] 7. Tamat D4<br>[ ] 8. Tamat S1<br>[ ] 9. Lebih tinggi dari S1<br>[ ] 88. Tidak yakin<br>[ ] 99. Menolak untuk menjawab |
| A7 | Pekerjaan ibu | [ ] 1. Ibu rumah tangga/ pensiunan<br>[ ] 2. PNS<br>[ ] 3. Pegawai swasta<br>[ ] 4. Pedagang kecil<br>[ ] 5. Pemilik bisnis / pengusaha<br>[ ] 6. Buruh<br>[ ] 7. Bertani/ nelayan<br>[ ] 8. Profesional mandiri (pengacara, arsitek, lainnya)<br>[ ] 9. Tidak bekerja<br>[ ] 99. Menolak untuk menjawab<br>[ ] 0. Meninggal (***<u>Lanjut ke pertanyaan A9</u>***) |
| A8 | Mothers' education | [ ] 1. Tidak pernah sekolah<br>[ ] 2. Tamat SD<br>[ ] 3. Tamat SMP<br>[ ] 4. Tamat SMA<br>[ ] 5. Tamat kejuruan<br>[ ] 6. Tamat D3<br>[ ] 7. Tamat D4<br>[ ] 8. Tamat S1<br>[ ] 9. Lebih tinggi dari S1<br>[ ] 88. Tidak yakin<br>[ ] 99. Menolak untuk menjawab |
| A9 | Pendapatan per bulan rumah tangga | [ ] 1. < Rp. 1.000.000<br>[ ] 2. Rp. 1.000.000 hingga Rp. 2.000.000<br>[ ] 3. Rp. 2.000.001 hingga Rp. 3.000.000<br>[ ] 4. Rp. 3.000.001 hingga Rp. 4.000.000<br>[ ] 5. Rp. 4.000.001 hingga Rp. 5.000.000<br>[ ] 6. Rp. 5.000.001 hingga Rp. 6.000.000<br>[ ] 7. > Rp. 6.000.000<br>[ ] 8. Tidak yakin / tidak yakin / tidak tahu<br>[ ] 9. Menolak untuk menjawab |
| A10 | Agama | [ ] 1. Islam<br>[ ] 2. Budha<br>[ ] 3. Kristen<br>[ ] 4. Lainnya<br>[ ] 9. Menolak untuk menjawab |
| A11 | Berapa kira-kira berat badan Anda (dalam kg.)? (kalau | [ ][ ][ ] |

| | | |
|---|---|---|
| | tidak tahu, tulis 888; kalau menolak untuk menjawab, tulis 999) | |
| A12 | Berapa kira-kira tinggi badan Anda (dalam cm.)? (kalau tidak tahu, tulis 888; kalau menolak untuk menjawab, tulis 999) | [ ][ ][ ] |
| A13 | Apa sumber media utama yang sering Anda temui untuk mendapatkan informasi tentang perilaku kesehatan? | [ ] 1. Iklan televisi<br>[ ] 2. Acara di masyarakat<br>[ ] 3. Brosur informasi<br>[ ] 4. Keluarga dan/atau teman<br>[ ] 5. Platform media sosial (seperti: Facebook, Twitter, Instagram, dan lainnya) |
| A14 | Ketika mendapatkan dan mencari informasi kesehatan, seberapa akurat menurut Anda informasinya? | [ ] 1. Sangat akurat<br>[ ] 2. Kadang-kadang akurat<br>[ ] 3. Sangat tidak akurat |
| A15 | Media sosial menyediakan sumber informasi yang bermanfaat | [ ] 1. Sangat setuju<br>[ ] 2. Setuju<br>[ ] 3. Tidak setuju<br>[ ] 4. Sangat tidak setuju |

## Bagian B. Paparan: Program promosi gizi "*Aksi Bergizi*"

| No | Pertanyaan | Jawaban |
|---|---|---|
| B1 | Apakah Anda ikut serta dalam kegiatan sesi Aksi Bergizi yang menunjukkan contoh makanan dan disesuaikan dengan kebutuhan nutrisi? | [ ] 0. Tidak (lanjut ke B3)<br>[ ] 1. Ya |
| B2 | Jika ya, apakah Anda menghadiri kegiatan-kegiatan berikut? (jawaban boleh lebih dari satu) | [ ] 1. Pengantar<br>[ ] 2. Aktifitas<br>[ ] 3. Diskusi |
| B3 | Apakah Anda ikut serta dalam kegiatan sesi Aksi Bergizi mengenai kebersihan pribadi dan beberapa contoh tindakan yang dilakukan? | [ ] 0. Tidak (lanjut ke B5)<br>[ ] 1. Ya |
| B4 | Jika ya, apakah Anda menghadiri kegiatan-kegiatan berikut? (jawaban boleh lebih dari satu) | [ ] 1. Pengantar<br>[ ] 2. Aktifitas<br>[ ] 3. Diskusi |
| B5 | Apakah Anda ikut serta dalam kegiatan sesi Aksi Bergizi mengenai mitos dan fakta tentang HIV serta menjadi subjek pembahasan tentang HIV? | [ ] 0. Tidak (lanjut ke B7)<br>[ ] 1. Ya |
| B6 | Jika ya, apakah Anda menghadiri kegiatan-kegiatan berikut? (jawaban boleh lebih dari satu) | [ ] 1. Pengantar<br>[ ] 2. Aktifitas<br>[ ] 3. Diskusi |
| B7 | Apakah Anda ikut serta dalam kegiatan sesi Aksi Bergizi mengenai zat adiktif dan menggunakan poster sebagai pemicu diskusi, selanjutnya mempresentasikan dengan drama sebagai bentuk proyeksi diri terkait remaja yang rentan menjadi pengguna narkoba? | [ ] 0. Tidak (lanjut ke B9)<br>[ ] 1. Ya |
| B8 | Jika ya, apakah Anda menghadiri kegiatan-kegiatan berikut? (jawaban boleh lebih dari satu) | [ ] 1. Pengantar<br>[ ] 2. Aktifitas<br>[ ] 3. Diskusi |
| B9 | Apakah Anda ikut serta dalam kegiatan sesi Aksi Bergizi mengenai Penyakit Tidak Menular (PTM), dan para peserta dapat mengenal PTM lebih ilmiah? | [ ] 0. Tidak (lanjut ke B11)<br>[ ] 1. Ya |
| B10 | Jika ya, apakah Anda menghadiri kegiatan-kegiatan berikut? (jawaban boleh lebih dari satu) | [ ] 1. Pengantar<br>[ ] 2. Aktifitas<br>[ ] 3. Diskusi |
| B11 | Apakah Anda ikut serta dalam kegiatan sesi Aksi Bergizi mengenai berbagai contoh aktivitas fisik, dan menghitung | [ ] 0. Tidak (lanjut ke B13)<br>[ ] 1. Ya |

| | | |
|---|---|---|
| | berbagai aktivitas fisik yang efektif serta peserta dapat lakukan dengan mudah setiap hari? | |
| B12 | Jika ya, apakah Anda menghadiri kegiatan-kegiatan berikut? (jawaban boleh lebih dari satu) | [ ] 1. Pengantar<br>[ ] 2. Aktifitas<br>[ ] 3. Diskusi |
| B13 | Dari opsi di bawah ini, mana yang dapat menggambarkan bahwa Anda ikut serta dalam kegiatan-kegiatan tersebut? | [ ] 1. Penuh perhatian (memperhatikan dengan seksama, melihat, dan mendengarkan semuanya).<br>[ ] 2. Relatif tinggi (berbicara sesekali dengan teman, tetapi melihat, dan mendengarkan semuanya). |

## Bagian C. Pendorong perilaku untuk pola makan dan kesehatan

| No | Pertanyaan | Jawaban |
|---|---|---|
| **Bias kognitif** | | |
| C1 | Saya mudah dipengaruhi oleh informasi nutrisi yang saya temui ketika memilih makanan | [ ] 1. Sangat tidak setuju<br>[ ] 2. Tidak setuju<br>[ ] 3. Setuju<br>[ ] 4. Sangat setuju |
| C2 | Sejauh mana Anda dipengaruhi oleh iklan atau informasi terkait kesehatan, yang mungkin membuat Anda membuat pilihan yang tidak sejalan dengan kepentingan terbaik Anda? | [ ] 1. Sangat tidak setuju<br>[ ] 2. Tidak setuju<br>[ ] 3. Setuju<br>[ ] 4. Sangat setuju |
| C3 | Seberapa sering Anda mencari informasi atau berita yang mengkonfirmasi keyakinan Anda bahwa merokok berbahaya bagi kesehatan? | [ ] 1. Tidak pernah<br>[ ] 2. Jarang<br>[ ] 3. Kadang-kadang<br>[ ] 4. Sebagian besar waktu<br>[ ] 5. Selalu |
| C4 | Seberapa sering Anda meremehkan risiko kesehatan dari konsumsi alkohol berdasarkan kenyataan bahwa Anda mengenal orang-orang yang minum secara teratur tanpa terlihat ada dampak negatif? | [ ] 1. Tidak pernah<br>[ ] 2. Jarang<br>[ ] 3. Kadang-kadang<br>[ ] 4. Sebagian besar waktu<br>[ ] 5. Selalu |
| C5 | Seberapa sering Anda membiarkan pengaruh sosial atau dari teman-teman memengaruhi keputusan Anda untuk tidak mengonsumsi zat-zat tertentu, misalnya, merasa tertekan untuk menggunakan zat-zat tersebut dalam situasi sosial tertentu? | [ ] 1. Tidak pernah<br>[ ] 2. Jarang<br>[ ] 3. Kadang-kadang<br>[ ] 4. Sebagian besar waktu<br>[ ] 5. Selalu |
| C6 | Sejauh mana Anda tidak beraktifitas fisik dibandingkan dengan potensi manfaat kesehatan jangka panjang yang diberikan? | [ ] 1. Tidak pernah<br>[ ] 2. Jarang<br>[ ] 3. Kadang-kadang |

| | | |
|---|---|---|
| | | [ ] 4. Sebagian besar waktu<br>[ ] 5. Selalu |
| **Minat** | | |
| C7 | Seberapa besar minat Anda untuk mempelajari dampak pilihan makanan terhadap kesehatan dan kesejahteraan secara keseluruhan? | [ ] 1. Tidak sama sekali tertarik<br>[ ] 2. Tidak terlalu tertarik<br>[ ] 3. Agak tertarik<br>[ ] 4. Sangat tertarik |
| C8 | Seberapa antusias Anda tentang potensi manfaat kesehatan dan peningkatan kualitas hidup yang datang dengan tidak merokok? | [ ] 1. Tidak sama sekali antusias<br>[ ] 2. Tidak terlalu antusias<br>[ ] 3. Agak antusias<br>[ ] 4. Sangat antusias |
| C9 | Seberapa termotivasi Anda untuk mengurangi atau menghentikan konsumsi alkohol demi kesehatan dan kesejahteraan Anda? | [ ] 1. Tidak sama sekali termotivasi<br>[ ] 2. Tidak terlalu termotivasi<br>[ ] 3. Agak termotivasi<br>[ ] 4. Sangat termotivasi |
| C10 | Seberapa bersedia Anda untuk mencari dukungan dan sumber daya untuk membantu Anda menolak godaan menggunakan narkoba? | [ ] 1. Tidak bersedia sama sekali<br>[ ] 2. Tidak terlalu bersedia<br>[ ] 3. Agak bersedia<br>[ ] 4. Sangat bersedia |
| C11 | Seberapa rasa ingin tahu Anda tentang manfaat dan perbaikan yang bisa Anda rasakan dalam hidup dengan menjadi aktif berolahraga? | [ ] 1. Tidak ingin tahu sama sekali<br>[ ] 2. Tidak terlalu ingin tahu<br>[ ] 3. Agak ingin tahu<br>[ ] 4. Sangat ingin tahu |
| C12 | Seberapa besar minat Anda untuk mempelajari metode dan praktik untuk melindungi kesehatan reproduksi Anda? | [ ] 1. Tidak sama sekali tertarik<br>[ ] 2. Tidak terlalu tertarik<br>[ ] 3. Agak tertarik<br>[ ] 4. Sangat tertarik |
| **Sikap** | | |
| C13 | Seberapa mungkin Anda setuju bahwa pola makan sehat dapat berkontribusi pada kesehatan secara keseluruhan dan mencegah penyakit? | [ ] 1. Sangat tidak setuju<br>[ ] 2. Tidak setuju<br>[ ] 3. Setuju<br>[ ] 4. Sangat setuju |
| C14 | Sejauh mana Anda setuju bahwa tidak merokok penting untuk kesehatan Anda? | [ ] 1. Sangat tidak setuju<br>[ ] 2. Tidak setuju<br>[ ] 3. Setuju<br>[ ] 4. Sangat setuju |
| C15 | Seberapa percaya diri Anda dalam kemampuan untuk menolak godaan untuk mengonsumsi alkohol? | [ ] 1. Sangat tidak percaya<br>[ ] 2. Tidak percaya<br>[ ] 3. Percaya<br>[ ] 4. Sangat percaya |
| C16 | Seberapa menyenangkan menurut Anda gaya hidup dengan tidak mengkonsumsi narkoba dibandingkan dengan mengonsumsi narkoba? | [ ] 1. Tidak menyenangkan<br>[ ] 2. Sedikit menyenangkan<br>[ ] 3. Cukup menyenangkan |

| | | |
|---|---|---|
| | | [ ] 4. Sangat menyenangkan |
| C17 | Seberapa penting bagi Anda untuk mengintegrasikan aktivitas fisik secara teratur dalam rutinitas harian Anda? | [ ] 1. Tidak penting<br>[ ] 2. Sedikit penting<br>[ ] 3. Cukup penting<br>[ ] 4. Sangat penting |
| C18 | Seberapa setuju Anda untuk mencari pemeriksaan rutin dan skrining untuk memastikan kesehatan reproduksi Anda dalam kondisi baik? | [ ] 1. Sangat tidak setuju<br>[ ] 2. Tidak setuju<br>[ ] 3. Setuju<br>[ ] 4. Sangat setuju |
| **Efikasi diri** | | |
| C19 | Saya yakin bahwa saya dapat menjaga pola makan yang sehat dan seimbang yang mencakup buah-buahan dan sayuran. | [ ] 1. Sangat tidak setuju<br>[ ] 2. Tidak setuju<br>[ ] 3. Setuju<br>[ ] 4. Sangat setuju |
| C20 | Saya yakin bahwa saya dapat membatasi konsumsi minuman ber gula (misalnya, minuman bersoda, minuman energi) menjadi satu kali seminggu atau kurang. | [ ] 1. Sangat tidak setuju<br>[ ] 2. Tidak setuju<br>[ ] 3. Setuju<br>[ ] 4. Sangat setuju |
| C21 | Saya yakin bahwa saya dapat menolak godaan untuk terlibat dalam perilaku yang tidak sehat (misalnya, ngemil berlebihan, menghindari olahraga). | [ ] 1. Sangat tidak setuju<br>[ ] 2. Tidak setuju<br>[ ] 3. Setuju<br>[ ] 4. Sangat setuju |
| C22 | Saya yakin bahwa saya dapat berlari (jogging) tiga hari atau lebih dalam seminggu daripada bersepeda dengan kecepatan biasa. | [ ] 1. Sangat tidak setuju<br>[ ] 2. Tidak setuju<br>[ ] 3. Setuju<br>[ ] 4. Sangat setuju |
| C23 | Saya yakin bahwa saya dapat berjalan kaki lima hari atau lebih dalam seminggu daripada duduk atau berbaring sambil menonton televisi. | [ ] 1. Sangat tidak setuju<br>[ ] 2. Tidak setuju<br>[ ] 3. Setuju<br>[ ] 4. Sangat setuju |
| C24 | Saya yakin bahwa saya dapat berhenti merokok (jika perokok) dan tetap bebas dari asap rokok. | [ ] 1. Sangat tidak setuju<br>[ ] 2. Tidak setuju<br>[ ] 3. Setuju<br>[ ] 4. Sangat setuju |
| C25 | Saya yakin bahwa saya dapat mengelola waktu saya dengan efektif untuk memiliki prioritas dan terlibat dalam perilaku sehat secara teratur. | [ ] 1. Sangat tidak setuju<br>[ ] 2. Tidak setuju<br>[ ] 3. Setuju<br>[ ] 4. Sangat setuju |
| C26 | Saya yakin bahwa saya dapat mencari dukungan sosial dan terlibat dalam kegiatan sehat bersama teman atau anggota keluarga. | [ ] 1. Sangat tidak setuju<br>[ ] 2. Tidak setuju<br>[ ] 3. Setuju<br>[ ] 4. Sangat setuju |
| C27 | Saya yakin bahwa saya dapat mengatasi | [ ] 1. Sangat tidak setuju |

| | | |
|---|---|---|
| | stres atau emosi negatif tanpa bergantung pada alkohol dan penggunaan zat sebagai mekanisme penanganan. | [ ] 2. Tidak setuju<br>[ ] 3. Setuju<br>[ ] 4. Sangat setuju |
| **Niat** | | |
| C28 | Seberapa tekad Anda untuk membuat perubahan dalam pola makan dan perilaku kesehatan Anda? | [ ] 1. Sangat tidak kuat<br>[ ] 2. Tidak kuat<br>[ ] 3. Kuat<br>[ ] 4. Sangat kuat |
| C29 | Seberapa mungkin Anda untuk secara aktif menerapkan perilaku makan dan kesehatan yang sehat ke dalam kehidupan sehari-hari Anda? | [ ] 1. Sangat tidak mungkin<br>[ ] 2. Tidak mungking<br>[ ] 3. Mungkin<br>[ ] 4. Sangat mungkin |
| C30 | Seberapa komitmen Anda untuk membuat perubahan jangka panjang dalam pola makan dan perilaku kesehatan Anda? | [ ] 1. Tidak komitmen<br>[ ] 2. Sedikit komitmen<br>[ ] 3. Cukup komitmen<br>[ ] 4. Sangat komitmen |
| **Rasionalitas terbatas** | | |
| C31 | Sejauh mana Anda menyederhanakan pemikiran saat membuat keputusan mengenai pola makan dan perilaku kesehatan Anda? | [ ] 1. Tidak pernah<br>[ ] 2. Jarang<br>[ ] 3. Kadang-kadang<br>[ ] 4. Sebagian besar waktu<br>[ ] 5. Selalu |
| C32 | Seberapa sering Anda cenderung membuat keputusan mengenai pola makan dan perilaku kesehatan Anda berdasarkan informasi terbatas atau pemahaman yang belum lengkap? | [ ] 1. Tidak pernah<br>[ ] 2. Jarang<br>[ ] 3. Kadang-kadang<br>[ ] 4. Sebagian besar waktu<br>[ ] 5. Selalu |
| C33 | Apakah Anda merasa sulit untuk mempertimbangkan semua opsi yang tersedia dan membuat pilihan yang tepat berdasarkan informasi mengenai pola makan dan perilaku kesehatan Anda? | [ ] 1. Tidak pernah<br>[ ] 2. Jarang<br>[ ] 3. Kadang-kadang<br>[ ] 4. Sebagian besar waktu<br>[ ] 5. Selalu |

## Bagian D. Kebiasaan diet

| No | Pertanyaan | Jawaban (Silahkan dicentang) |
|---|---|---|
| catatan_ffq | **Kuesioner Frekuensi Konsumsi Makanan** Seberapa sering Anda makan makanan berikut? | |
| D1 | Beras (beras putih) | [ ] 1. Tidak pernah atau kurang dari satu kali per bulan<br>[ ] 2. 1-3 kali per bulan<br>[ ] 3. Satu kali per minggu<br>[ ] 4. 2-4 kali per minggu<br>[ ] 5. 5-6 kali per minggu<br>[ ] 6. 1 kali sehari<br>[ ] 7. Lebih dari satu kali sehari<br>[ ] 99. Menolak untuk menjawab |
| D2 | Produk gandum olahan (roti tawar, mie) | [ ] 1. Tidak pernah atau kurang dari satu kali per bulan<br>[ ] 2. 1-3 kali per bulan<br>[ ] 3. Satu kali per minggu<br>[ ] 4. 2-4 kali per minggu<br>[ ] 5. 5-6 kali per minggu<br>[ ] 6. 1 kali sehari<br>[ ] 7. Lebih dari satu kali sehari<br>[ ] 99. Menolak untuk menjawab |
| D3 | Beras merah | [ ] 1. Tidak pernah atau kurang dari satu kali per bulan<br>[ ] 2. 1-3 kali per bulan<br>[ ] 3. Satu kali per minggu<br>[ ] 4. 2-4 kali per minggu<br>[ ] 5. 5-6 kali per minggu<br>[ ] 6. 1 kali sehari<br>[ ] 7. Lebih dari satu kali sehari<br>[ ] 99. Menolak untuk menjawab |
| D4 | Produk gandum utuh (misalnya, roti gandum, mie gandum) | [ ] 1. Tidak pernah atau kurang dari satu kali per bulan<br>[ ] 2. 1-3 kali per bulan<br>[ ] 3. Satu kali per minggu<br>[ ] 4. 2-4 kali per minggu<br>[ ] 5. 5-6 kali per minggu<br>[ ] 6. 1 kali sehari<br>[ ] 7. Lebih dari satu kali sehari<br>[ ] 99. Menolak untuk menjawab |

| No | Pertanyaan | Jawaban (Silahkan dicentang) |
| --- | --- | --- |
| D5 | Umbi-umbian (singkong, talas, ubi jalar putih, kentang) | [ ] 1. Tidak pernah atau kurang dari satu kali per bulan<br>[ ] 2. 1-3 kali per bulan<br>[ ] 3. Satu kali per minggu<br>[ ] 4. 2-4 kali per minggu<br>[ ] 5. 5-6 kali per minggu<br>[ ] 6. 1 kali sehari<br>[ ] 7. Lebih dari satu kali sehari<br>[ ] 99. Menolak untuk menjawab |
| D6 | Daging (sapi, domba) | [ ] 1. Tidak pernah atau kurang dari satu kali per bulan<br>[ ] 2. 1-3 kali per bulan<br>[ ] 3. Satu kali per minggu<br>[ ] 4. 2-4 kali per minggu<br>[ ] 5. 5-6 kali per minggu<br>[ ] 6. 1 kali sehari<br>[ ] 7. Lebih dari satu kali sehari<br>[ ] 99. Menolak untuk menjawab |
| D7 | Produk ungags (bebek, ayam) | [ ] 1. Tidak pernah atau kurang dari satu kali per bulan<br>[ ] 2. 1-3 kali per bulan<br>[ ] 3. Satu kali per minggu<br>[ ] 4. 2-4 kali per minggu<br>[ ] 5. 5-6 kali per minggu<br>[ ] 6. 1 kali sehari<br>[ ] 7. Lebih dari satu kali sehari<br>[ ] 99. Menolak untuk menjawab |
| D8a | Ikan (mentah, panggang, sup, tidak digoreng) | [ ] 1. Tidak pernah atau kurang dari satu kali per bulan<br>[ ] 2. 1-3 kali per bulan<br>[ ] 3. Satu kali per minggu<br>[ ] 4. 2-4 kali per minggu<br>[ ] 5. 5-6 kali per minggu<br>[ ] 6. 1 kali sehari<br>[ ] 7. Lebih dari satu kali sehari<br>[ ] 99. Menolak untuk menjawab |
| D8b | Produk laut segar (misalnya, kerang, udang, kepiting, gurita) | [ ] 1. Tidak pernah atau kurang dari satu kali per bulan<br>[ ] 2. 1-3 kali per bulan<br>[ ] 3. Satu kali per minggu<br>[ ] 4. 2-4 kali per minggu<br>[ ] 5. 5-6 kali per minggu<br>[ ] 6. 1 kali sehari<br>[ ] 7. Lebih dari satu kali sehari |

| No | Pertanyaan | Jawaban (Silahkan dicentang) |
|---|---|---|
| | | [ ] 99. Menolak untuk menjawab |
| D9 | Telur | [ ] 1. Tidak pernah atau kurang dari satu kali per bulan<br>[ ] 2. 1-3 kali per bulan<br>[ ] 3. Satu kali per minggu<br>[ ] 4. 2-4 kali per minggu<br>[ ] 5. 5-6 kali per minggu<br>[ ] 6. 1 kali sehari<br>[ ] 7. Lebih dari satu kali sehari<br>[ ] 99. Menolak untuk menjawab |
| D10 | Sayuran berdaun hijau (misalnya, sawi hijau, kacang panjang, kale, bayam, sawi, timun) | [ ] 1. Tidak pernah atau kurang dari satu kali per bulan<br>[ ] 2. 1-3 kali per bulan<br>[ ] 3. Satu kali per minggu<br>[ ] 4. 2-4 kali per minggu<br>[ ] 5. 5-6 kali per minggu<br>[ ] 6. 1 kali sehari<br>[ ] 7. Lebih dari satu kali sehari<br>[ ] 99. Menolak untuk menjawab |
| D11 | Sayuran berwarna kuning atau oranye (misalnya, labu, ubi jalar, wortel, pepaya matang) | [ ] 1. Tidak pernah atau kurang dari satu kali per bulan<br>[ ] 2. 1-3 kali per bulan<br>[ ] 3. Satu kali per minggu<br>[ ] 4. 2-4 kali per minggu<br>[ ] 5. 5-6 kali per minggu<br>[ ] 6. 1 kali sehari<br>[ ] 7. Lebih dari satu kali sehari<br>[ ] 99. Menolak untuk menjawab |
| D12 | Produk kedelai (misalnya., tahu, tempe) | [ ] 1. Tidak pernah atau kurang dari satu kali per bulan<br>[ ] 2. 1-3 kali per bulan<br>[ ] 3. Satu kali per minggu<br>[ ] 4. 2-4 kali per minggu<br>[ ] 5. 5-6 kali per minggu<br>[ ] 6. 1 kali sehari<br>[ ] 7. Lebih dari satu kali sehari<br>[ ] 99. Menolak untuk menjawab |
| D13 | Sayuran yang diawetkan (misalnya, sayuran kaleng) | [ ] 1. Tidak pernah atau kurang dari satu kali per bulan<br>[ ] 2. 1-3 kali per bulan<br>[ ] 3. Satu kali per minggu<br>[ ] 4. 2-4 kali per minggu<br>[ ] 5. 5-6 kali per minggu |

| No | Pertanyaan | Jawaban (Silahkan dicentang) |
|---|---|---|
| | | [ ] 6. 1 kali sehari<br>[ ] 7. Lebih dari satu kali sehari<br>[ ] 99. Menolak untuk menjawab |
| D14 | Buah segar | [ ] 1. Tidak pernah atau kurang dari satu kali per bulan<br>[ ] 2. 1-3 kali per bulan<br>[ ] 3. Satu kali per minggu<br>[ ] 4. 2-4 kali per minggu<br>[ ] 5. 5-6 kali per minggu<br>[ ] 6. 1 kali sehari<br>[ ] 7. Lebih dari satu kali sehari<br>[ ] 99. Menolak untuk menjawab |
| D15 | Produk susu (susu segar, susu bubuk, susu kemasan) | [ ] 1. Tidak pernah atau kurang dari satu kali per bulan<br>[ ] 2. 1-3 kali per bulan<br>[ ] 3. Satu kali per minggu<br>[ ] 4. 2-4 kali per minggu<br>[ ] 5. 5-6 kali per minggu<br>[ ] 6. 1 kali sehari<br>[ ] 7. Lebih dari satu kali sehari<br>[ ] 99. Menolak untuk menjawab |
| D16 | Makanan ringan kemasan (misalnya, chiki jagung, keripik kentang Lay) | [ ] 1. Tidak pernah atau kurang dari satu kali per bulan<br>[ ] 2. 1-3 kali per bulan<br>[ ] 3. Satu kali per minggu<br>[ ] 4. 2-4 kali per minggu<br>[ ] 5. 5-6 kali per minggu<br>[ ] 6. 1 kali sehari<br>[ ] 7. Lebih dari satu kali sehari<br>[ ] 99. Menolak untuk menjawab |
| D17 | Makanan ringan tanpa kemasan (donat, kentang goreng, roti, dll.) | [ ] 1. Tidak pernah atau kurang dari satu kali per bulan<br>[ ] 2. 1-3 kali per bulan<br>[ ] 3. Satu kali per minggu<br>[ ] 4. 2-4 kali per minggu<br>[ ] 5. 5-6 kali per minggu<br>[ ] 6. 1 kali sehari<br>[ ] 7. Lebih dari satu kali sehari<br>[ ] 99. Menolak untuk menjawab |
| D18 | Minuman atau yang diberi pemanis (susu kedelai, minuman ringan, cola, kopi atau teh dengan gula, susu | [ ] 1. Tidak pernah atau kurang dari satu kali per bulan<br>[ ] 2. 1-3 kali per bulan<br>[ ] 3. Satu kali per minggu |

| No | Pertanyaan | Jawaban (Silahkan dicentang) |
|---|---|---|
| | kental manis) | [ ] 4. 2-4 kali per minggu<br>[ ] 5. 5-6 kali per minggu<br>[ ] 6. 1 kali sehari<br>[ ] 7. Lebih dari satu kali sehari<br>[ ] 99. Menolak untuk menjawab |
| D19 | Makanan olahan atau ultraproses (ikan kaleng, sosis, sayuran kaleng, makanan beku, dll.) | [ ] 1. Tidak pernah atau kurang dari satu kali per bulan<br>[ ] 2. 1-3 kali per bulan<br>[ ] 3. Satu kali per minggu<br>[ ] 4. 2-4 kali per minggu<br>[ ] 5. 5-6 kali per minggu<br>[ ] 6. 1 kali sehari<br>[ ] 7. Lebih dari satu kali sehari<br>[ ] 99. Menolak untuk menjawab |
| D20 | Makanan penutup (misalnya, es krim, kue, permen, kue kering, ketan, makanan manis lainnya) | [ ] 1. Tidak pernah atau kurang dari satu kali per bulan<br>[ ] 2. 1-3 kali per bulan<br>[ ] 3. Satu kali per minggu<br>[ ] 4. 2-4 kali per minggu<br>[ ] 5. 5-6 kali per minggu<br>[ ] 6. 1 kali sehari<br>[ ] 7. Lebih dari satu kali sehari<br>[ ] 99. Menolak untuk menjawab |
| D21 | Daging atau ikan yang digoreng (ikan goreng, ayam goreng, daging sapi goreng) | [ ] 1. Tidak pernah atau kurang dari satu kali per bulan<br>[ ] 2. 1-3 kali per bulan<br>[ ] 3. Satu kali per minggu<br>[ ] 4. 2-4 kali per minggu<br>[ ] 5. 5-6 kali per minggu<br>[ ] 6. 1 kali sehari<br>[ ] 7. Lebih dari satu kali sehari<br>[ ] 99. Menolak untuk menjawab |

## Bagian E. Kebersihan Pribadi

Silakan jawab sejauh mana Anda mempraktikkan pernyataan-pernyataan berikut?

| No | Pertanyaan | Jawaban |
|---|---|---|
| E1 | Selama 30 hari terakhir, seberapa sering Anda mencuci tangan sebelum makan? Silakan jawab sejauh mana Anda mempraktikkan pernyataan-pernyataan berikut? | [ ] 1. Tidak pernah<br>[ ] 2. Jarang<br>[ ] 3. Kadang-kadang<br>[ ] 4. Sebagian besar waktu<br>[ ] 5. Selalu |
| E2 | Selama 30 hari terakhir, seberapa sering | [ ] 1. Tidak pernah |

| | | |
|---|---|---|
| | Anda mencuci tangan setelah menggunakan toilet atau bak mandi? | [ ] 2. Jarang<br>[ ] 3. Kadang-kadang<br>[ ] 4. Sebagian besar waktu<br>[ ] 5. Selalu |
| E3 | Selama 30 hari terakhir, seberapa sering Anda menggunakan sabun saat mencuci tangan? | [ ] 1. Tidak pernah<br>[ ] 2. Jarang<br>[ ] 3. Kadang-kadang<br>[ ] 4. Sebagian besar waktu<br>[ ] 5. Selalu |
| E4 | Selama 30 hari terakhir, berapa kali sehari biasanya Anda membersihkan atau menyikat gigi? | [ ] 1. Tidak pernah<br>[ ] 2. Tidak setiap hari<br>[ ] 3. Setiap hari, sekali sehari<br>[ ] 4. Setiap hari, dua kali sehari<br>[ ] 5. Setiap hari, lebih dari dua kali sehari |
| E5 | Selama 30 hari terakhir, seberapa sering Anda menggunakan pasta gigi yang mengandung fluoride saat membersihkan atau menyikat gigi? | [ ] 1. Tidak pernah<br>[ ] 2. Tidak setiap hari<br>[ ] 3. Setiap hari, sekali sehari<br>[ ] 4. Setiap hari, dua kali sehari<br>[ ] 5. Setiap hari, lebih dari dua kali sehari |
| E6 | Selama 30 hari terakhir, seberapa sering Anda memotong kuku? | [ ] 1. Tidak pernah<br>[ ] 2. Satu kali per minggu<br>[ ] 3. Satu kali per dua minggu<br>[ ] 4. Satu kali per tiga minggu<br>[ ] 5. Satu kali per 4 minggu atau tidak memotong kuku |

## Bagian F. Persepsi Penyakit Tidak Menular (PTM)

Silakan pilih sejauh mana Anda setuju dengan pernyataan-pernyataan berikut:

| No | Pertanyaan | Sangat setuju | Setuju | Tidak setuju | Sangat tidak setuju |
|---|---|---|---|---|---|
| F1 | Menurut saya, penyakit tidak menular melibatkan kondisi kesehatan jangka panjang | | | | |
| F2 | Penyakit tidak menular mencakup kondisi seperti diabetes, penyakit jantung, dan kanker. | | | | |
| F3 | Saya merasa bahwa saya dapat dengan efektif memberi tahu orangtua untuk tidak | | | | |

| | | | | | |
|---|---|---|---|---|---|
| | merokok | | | | |
| F4 | Saya merasa bahwa saya dapat mengingatkan anggota keluarga saya untuk menjaga berat badan yang ideal | | | | |
| F5 | Saya merasa bahwa saya dapat mengingatkan anggota keluarga saya untuk berolahraga secara rutin | | | | |
| F6 | Saya merasa bahwa saya dapat dengan efektif mengingatkan anggota keluarga saya untuk mengonsumsi pola makan sehat dan seimbang (atau) menjaga pola makan yang sehat dan seimbang guna mencegah Penyakit Tidak Menular (PTM). | | | | |
| F7 | Saya merasa bahwa saya dapat dengan efektif mengingatkan anggota keluarga saya untuk mengonsumsi obat sesuai resep secara teratur, jika mereka memiliki hipertensi, hiperlipidemia, atau diabetes | | | | |

F8. Menurut pendapat Anda, bagaimana para pemuda dapat membantu Indonesia melawan Penyakit Tidak Menular (PTM)?

**[……………………………………………………………………………]**

# Bagian G. Aktifitas fisik

Pikirkan semua kegiatan berenergi yang Anda lakukan dalam 7 hari terakhir. Kegiatan fisik berenergi tinggi merujuk pada kegiatan yang memerlukan usaha fisik keras dan membuat Anda bernapas lebih keras dari biasanya. Pikirkan hanya tentang kegiatan fisik tersebut yang Anda lakukan setidaknya selama 10 menit sekaligus?

G1. Selama 7 hari terakhir, berapa hari Anda melakukan kegiatan fisik berenergi tinggi seperti mengangkat beban berat, menggali, aerobik, atau bersepeda cepat?

_____ hari per minggu (rentang 1-7)

[ ] Tidak ada aktifitas fisik berenergi tinggi → **Lanjut ke pertanyaan G3**

G2. Berapa lama waktu yang biasanya Anda habiskan untuk melakukan kegiatan fisik berenergi tinggi pada salah satu dari hari tersebut?

_____ jam per hari

_____ menit per hari

[ ] Tidak tahu/ tidak yakin

Pikirkan semua kegiatan sedang yang Anda lakukan dalam 7 hari terakhir. Kegiatan sedang merujuk pada kegiatan yang memerlukan usaha fisik sedang dan membuat Anda bernapas agak lebih keras dari biasanya. Pikirkan hanya tentang kegiatan fisik tersebut yang Anda lakukan setidaknya selama 10 menit sekaligus.

G3. Selama 7 hari terakhir, berapa hari Anda melakukan kegiatan fisik sedang seperti membawa beban ringan, bersepeda dengan kecepatan biasa, atau tenis/badminton ganda? Jangan masukkan jalan kaki.

_____ hari per minggu (rentang 1-7)

[ ] Tidak ada aktifitas fisik sedang → **Lanjut ke pertanyaan G5**

G4. Berapa lama waktu yang biasanya Anda habiskan untuk melakukan kegiatan fisik sedang pada salah satu dari hari tersebut?

_____ jam per hari

_____ menit per hari

[ ] Tidak tahu/ tidak yakin

Pikirkan waktu yang Anda habiskan untuk berjalan dalam 7 hari terakhir. Ini mencakup berjalan di sekolah dan di rumah, berjalan untuk bepergian dari satu tempat ke tempat lain, dan setiap berjalan yang Anda lakukan semata-mata untuk rekreasi, olahraga, latihan, atau bersantai.

G5. Selama 7 hari terakhir, berapa hari Anda berjalan setidaknya selama 10 menit sekaligus?

_____ hari per minggu (rentang 1-7)

[ ] Tidak ada berjalan kaki → **Lanjut ke pertanyaan G7**

G6. Berapa lama waktu yang biasanya Anda habiskan untuk berjalan pada salah satu dari hari tersebut?

_____ jam per hari

_____ menit per hari

[ ] Tidak tahu/ tidak yakin

Pertanyaan terakhir adalah tentang waktu yang Anda habiskan untuk duduk pada hari kerja selama 7 hari terakhir. Termasuk waktu yang dihabiskan di sekolah, di rumah, saat mengerjakan tugas, dan selama waktu senggang. Ini dapat mencakup waktu yang dihabiskan

untuk duduk di meja, mengunjungi teman, membaca, atau duduk atau berbaring untuk menonton televisi.

G7. Selama 7 hari terakhir, berapa lama waktu yang Anda habiskan untuk duduk pada hari kerja?

_____ jam per hari

_____ menit per hari

[ ] Tidak tahu/ tidak yakin

## Bagian H. Penggunaan Alkohol, Tembakau, dan Zat Adiktif

H1. Menurut pendapat Anda, mengapa para pemuda menggunakan zat adiktif? Mengapa beberapa dari mereka yang menggunakan zat adiktif dan melakukannya secara berlebihan, hingga terjadi gangguan penggunaan zat?

**[..............................................................................]**

H2. Menurut pendapat Anda, bagaimana generasi muda dapat menghindari penggunaan zat adiktif?

**[..............................................................................]**

**<u>Petunjuk</u>** Mohon dapat dicentang ✓ **di ruang kosong yang sesuai dengan jawaban Anda**

| **Penggunaan alkohol** | |
|---|---|
| H3. Selama hidup Anda, pernahkah Anda minum alkohol (tidak termasuk mencicipi atau mencoba dalam jumlah yang sangat kecil)? | [ ] 0) Tidak pernah minum ***<u>(Lanjut ke pertanyaan H8)</u>***<br>[ ] 1) Ya<br>[ ] 88) Tidak yakin ***<u>(Lanjut ke pertanyaan H8)</u>***<br>[ ] 99) Menolak untuk menjawab ***<u>(Lanjut ke pertanyaan H8)</u>*** |
| H4. Kapan kamu mulai pertama kali minum alkohol? | Mulai minum umur...............tahun<br>[ ] 88) Tidak yakin<br>[ ] 99) Menolak untuk menjawab |
| H5. Kapan terakhir kali Anda minum (tidak termasuk mencicipi atau mencoba dalam jumlah yang sangat kecil)? | [ ] 1) Lebih dari 12 bulan yang lalu<br>[ ] 2) Lebih dari 30 hari yang lalu tetapi dalam 12 bulan terakhir.<br>[ ] 3) Lebih dari 1 minggu yang lalu tetapi |

| | |
|---|---|
| | dalam 30 hari terakhir.<br>[ ] 4) Dalam 1 minggu yang lalu |
| H6. Jika Anda ingin minum alkohol, berapa lama waktu yang dibutuhkan untuk membeli/mencari minuman? | Akan memakan waktu...................... menit<br>[ ] 77) Lebih dari 1 hari<br>[ ] 88) Tidak yakin<br>[ ] 99) Menolak untuk menjawab |
| H7. **Dalam 30 hari terakhir,** berapa kali Anda minum? | [ ] 0) Tidak pernah<br>[ ] 1) 1-2 kali<br>[ ] 2) 3-5 kali<br>[ ] 3) 6-9 kali<br>[ ] 4) 10-19 kali<br>[ ] 5) 20 kali atau lebih<br>[ ] 88) Tidak yakin<br>[ ] 99) Menolak untuk menjawab |
| **Penggunaan tembakau** | |
| H8. Selama hidup Anda, apakah Anda pernah merokok lebih dari 5 bungkus atau 100 batang rokok? | [ ] 0) Tidak pernah merokok ***(Lanjut ke pertanyaan H16)***<br>[ ] 1) Iya, tetapi tidak lebih dari 5 bungkus atau 100 batang.<br>[ ] 2) Ya<br>[ ] 88) Tidak yakin ***((Lanjut ke pertanyaan H16)***<br>[ ] 99) Menolak untuk menjawab ***((Lanjut ke pertanyaan H16)*** |
| H9. Berapa usia Anda ketika Anda mulai merokok merokok atau produk tembakau lainnya? | Mulai merokok umur..............tahun<br>[ ] 88) Tidak yakin<br>[ ] 99) Menolak untuk menjawab |
| H10. Kapan terakhir kali Anda merokok | [ ] 1) Lebih dari 12 bulan yang lalu ***((Lanjut ke pertanyaan H16)***<br>[ ] 2) Lebih dari 30 hari yang lalu tetapi dalam 12 bulan terakhir.<br>[ ] 3) Lebih dari 1 minggu yang lalu tetapi dalam 30 hari terakhir.<br>[ ] 4) Dalam 1 minggu yang lalu |
| H11. Jika Anda ingin merokok sebatang rokok, berapa lama waktu yang dibutuhkan untuk membeli/mencarinya? | Akan memakan waktu...................... menit<br>[ ] 77) Lebih dari 1 hari<br>[ ] 88) Tidak yakin<br>[ ] 99) Menolak untuk menjawab |
| H12. Dalam 12 bulan terakhir, pernahkah Anda mencoba berhenti merokok? | [ ] 0) Tidak<br>[ ] 1) Ya<br>[ ] 99) Menolak untuk menjawab |
| H13. Dalam 12 bulan terakhir, pernahkah Anda berhenti merokok? | [ ] 0) Tidak<br>[ ] 1) Ya, dalam ………….. hari<br>[ ] 99) Menolak untuk menjawab |
| H14. Dalam 30 hari terakhir, berapa hari | [ ] 0) Tidak ada ***(Lanjut ke pertanyaan*** |

| | |
|---|---|
| Anda merokok? | ***H16)***<br>[ ] 1) 1-2 hari<br>[ ] 2) 3-5 hari<br>[ ] 3) 6-9 hari<br>[ ] 4) 10-19 hari<br>[ ] 5) 20 hari atau lebih<br>[ ] 88) Tidak yakin<br>[ ] 99) Menolak untuk menjawab |
| H15. <u>Dalam 30 hari terakhir,</u> pada hari-hari ketika Anda merokok, berapa banyak rokok yang Anda gunakan? | [ ] 1) Kurang dari 1 per hari<br>[ ] 2) 1 per hari<br>[ ] 3) 2-5 per hari<br>[ ] 4) 6-10 per hari<br>[ ] 5) 11-20 per hari<br>[ ] 6) Lebih dari 20 per hari<br>[ ] 88) Tidak yakin<br>[ ] 99) Menolak untuk menjawab |
| H16. Selama hidup Anda, pernahkah Anda menggunakan rokok elektronik? | [ ] 0) Tidak pernah merokok ***(Lanjut ke pertanyaan H22)***<br>[ ] 1) Ya<br>[ ] 99) Menolak untuk menjawab ***(Lanjut ke pertanyaan H22)*** |
| H16a. Jika ya, berapa usia Anda ketika Anda pertama kali menggunakan rokok elektronik? | a) Saya memulai saat berumur ……………. tahun<br>[ ] 88) Tidak yakin<br>[ ] 99) Menolak untuk menjawab |
| H17. Kapan terakhir kali Anda menggunakan rokok elektronik? | [ ] 1) Lebih dari 12 bulan yang lalu<br>***((Lanjut ke pertanyaan H22)***<br>[ ] 2) Lebih dari 30 hari yang lalu tetapi dalam 12 bulan terakhir.<br>[ ] 3) Lebih dari 1 minggu yang lalu tetapi dalam 30 hari terakhir.<br>[ ] 4) Dalam 1 minggu yang lalu |
| H18. <u>Dalam 12 bulan terakhir, pernahkah Anda mencoba berhenti menggunakan rokok elektronik?</u> | [ ] 0) Tidak<br>[ ] 1) Ya<br>[ ] 99) Menolak untuk menjawab |
| H19. <u>Dalam 12 bulan terakhir, pernahkah Anda berhenti menggunakan rokok elektronik?</u> | [ ] 0) No<br>[ ] 1) Ya, dalam ………….. hari<br>[ ] 99) Menolak untuk menjawab |
| H20. <u>Dalam 30 hari terakhir, berapa hari Anda menggunakan rokok elektronik?</u> | [ ] 0) Tidak ada ***(Lanjut ke pertanyaan H22)***<br>[ ] 1) 1-2 hari<br>[ ] 2) 3-5 hari<br>[ ] 3) 6-9 hari<br>[ ] 4) 10-19 hari<br>[ ] 5) 20 hari atau lebih<br>[ ] 88) Tidak yakin |

| | |
|---|---|
| | [ ] 99) Menolak untuk menjawab |
| H21. <u>Dalam 30 hari terakhir, pada hari-hari ketika Anda menggunakan rokok elektronik, berapa kali per hari Anda menggunakan rokok elektronik? (satu "KALI" terdiri dari sekitar 15 hirupan)</u> | [ ] 1) Kurang dari 1 per hari<br>[ ] 2) 1 per hari<br>[ ] 3) 2-5 per hari<br>[ ] 4) 6-10 per hari<br>[ ] 5) 11-20 per hari<br>[ ] 6) Lebih dari 20 per hari<br>[ ] 88) Tidak yakin<br>[ ] 99) Menolak untuk menjawab |

**Penggunaan zat adiktif**

| **<u>H22. Apakah Anda pernah menggunakan obat atau zat berikut?</u>** | **1) Selama hidup Anda (kalau "Tidak", lanjut ke baris berikutnya) atau (kalau"Ya", lanjut ke kolom sebelah kanan)** | | **2) Dalam 12 bulan terakhir (kalau "Tidak", lanjut ke baris berikutnya) atau (kalau"Ya", lanjut ke kolom sebelah kanan)** | | **3) Dalam 30 hari terakhir** | |
|---|---|---|---|---|---|---|
| | **Tidak** | **Ya** | **Tidak** | **Ya** | **Tidak** | **Ya** |
| a. Analgesik (tidak sebagai obat) (*parasetamol*) | | | | | | |
| b. Antihistamine (tidak sebagai obat) (*cetirizine*) | | | | | | |
| c. Obat batuk (tidak sebagai obat) | | | | | | |
| d. Anxiolytics (*obat anti depresi*) | | | | | | |
| e. Sedatives (*obat penenang*) | | | | | | |
| f. Minuman berenergi (*extra joss, M-150, kratingdaeng, hemaviton, lainnya*) | | | | | | |
| g. *PRO* [procodyl, promethazine] (*obat anti mabok/ antimo*) | | | | | | |
| h. *Racikan dari obat demam dengan dosis tinggi yang dicampur dengan minuman soda, es, dan juga permen sebagai penambah rasa* | | | | | | |
| i. Poppers (*produk kimia yang mengandung zat Amyl nitrite yang digunakan dengan cara dihirup*) | | | | | | |
| j. *Daun kratom, ketum, purik, sepat, kedamba, ithang, kakuan, thom, atau biak)* | | | | | | |
| k. *Daun kratom, ketum, purik, sepat, kedamba, ithang, kakuan, thom, atau biak yang dicampur dengan zat lain*) | | | | | | |
| l. Cannabis (*Ganja*) | | | | | | |
| m. Opium (*Candu*) | | | | | | |

| | | | | | | |
|---|---|---|---|---|---|---|
| n. Ecstasy / Love Drug (*Inex*) | | | | | | |
| o. Ketamine (*K khusus*) | | | | | | |
| p. Heroin (*Putau*) | | | | | | |
| q. *Menghirup cairan tiner, lem, dan lainnya*) | | | | | | |
| r. Methamphetamine (*Sabu*) | | | | | | |
| s. Crystal methamphetamine (*Narkoba Kristal*) | | | | | | |

## Bagian I. Kesehatan Reproduksi dan Pencegahan Penyakit Menular Seksual (PMS)

Silakan pilih sejauh mana Anda setuju dengan pernyataan-pernyataan berikut:

| No | Pertanyaan | Sangat setuju | Setuju | Tidak setuju | Sangat tidak setuju |
|---|---|---|---|---|---|
| I1 | Saya tidak ingin berjabat tangan ketika menyapa seseorang yang terinfeksi HIV/AIDS. | | | | |
| I2 | Remaja yang HIV-positif seharusnya dilarang menghadiri sekolah dengan siswa HIV-negatif | | | | |
| I3 | Remaja yang HIV-positif seharusnya tidak berenang di kolam renang bersama siswa yang HIV-negatif. | | | | |
| I4 | Remaja yang HIV-positif seharusnya tidak ikut serta dalam kompetisi olahraga dengan siswa yang HIV-negatif. | | | | |

I5. Jika Anda memiliki teman yang HIV positif, Anda pergi berenang bersama mereka, dan seseorang mengatakan sesuatu yang menentang teman Anda menggunakan kolam renang. Apa yang akan Anda lakukan?

[……………………………………………………………………………]

**<u>Petunjuk</u>** Mohon dapat dicentang ✓ **di ruang kosong yang sesuai dengan jawaban Anda**

| | |
|---|---|
| I6a. Pernahkah Anda memiliki pasangan yang romantis? | [ ] 0) Tidak<br>[ ] 1) Ya<br>[ ] 9) Menolak untuk menjawab |
| I6b. Kepada jenis kelamin mana Anda tertarik? (boleh memilih lebih dari satu | [ ] 1) Laki-laki<br>[ ] 2) Perempuan |

| | |
|---|---|
| jawaban) | [ ] 3) Bencong<br>[ ] 4) Tomboy<br>[ ] 5) Tidak laki-laki maupun perempuan<br>[ ] 6) Saya tidak yakin, kepada siapa saya tertarik<br>[ ] 7) Saya tidak tertarik ke gender apapun<br>[ ] 9) Menolak untuk menjawab |
| I7. Metode seks aman atau pencegahan penyakit menular seksual apa yang Anda ketahui? (Diperbolehkan lebih dari 1 jawaban) | [ ] 0) Tidak ada<br>[ ] 1) Menahan diri<br>[ ] 2) Menghindari aktifitas berisiko tinggi dengan risiko robekan mukosa<br>[ ] 3) Menggunakan kondom<br>[ ] 4) Suppositoria<br>[ ] 5) Mengonsumsi profilaksis sebelum paparan (PreP)<br>[ ] 6) Mengonsumsi profilaksis setelah paparan (PEP)<br>[ ] 7) Metode lain, sebutkan.......................<br>[ ] 88) Tidak tahu / tidak yakin<br>[ ] 99) Menolak untuk menjawab |
| I8. Pernahkah Anda diajari tentang AIDS atau HIV di sekolah? | [ ] 0) Tidak pernah<br>[ ] 1) Ya<br>[ ] 8) Tidak tahu/ tidak yakin<br>[ ] 9) Menolak untuk menjawab |
| I9. Pernahkah Anda diajari tentang cara mengontrol kehamilan di sekolah? | [ ] 0) Tidak pernah<br>[ ] 1) Ya<br>[ ] 8) Tidak tahu/ tidak yakin<br>[ ] 9) Menolak untuk menjawab |
